# Drivers of Regional Bacterial Community Structure and Diversity in the Northwest Atlantic Ocean

**DOI:** 10.3389/fmicb.2019.00281

**Published:** 2019-02-21

**Authors:** Jackie Zorz, Ciara Willis, André M. Comeau, Morgan G. I. Langille, Catherine L. Johnson, William K. W. Li, Julie LaRoche

**Affiliations:** ^1^Department of Biology, Dalhousie University, Halifax, NS, Canada; ^2^Department of Geoscience, University of Calgary, Calgary, AB, Canada; ^3^CGEB-Integrated Microbiome Resource, Department of Pharmacology, Dalhousie University, Halifax, NS, Canada; ^4^Fisheries and Oceans Canada, Bedford Institute of Oceanography, Dartmouth, NS, Canada

**Keywords:** Northwest Atlantic, microbial ecology, biogeography, oceans and seas, 16S rRNA, Scotian Shelf, marine microbiology, bacterial diversity

## Abstract

The fundamental role of bacteria in global biogeochemical cycles warrants a thorough understanding of the factors controlling bacterial community structure. In this study, the integrated effect of seasonal differences and spatial distribution on bacterial community structure and diversity were investigated at the regional scale. We conducted a comprehensive bacterial survey, with 451 samples of the Scotian Shelf sector of the Northwest Atlantic Ocean during spring and fall of 2014 and 2016, to analyze the effects of physicochemical gradients on bacterial community structure. Throughout the region, Pelagibacteraceae and Rhodobacteraceae were the most common in the free-living fraction, while Flavobacteriia and Deltaproteobacteria were more abundant in the particle-associated fraction. Overall, there was strong covariation of the microbial community diversity from the two size fractions. This relationship existed despite the statistically significant difference in community structure between the free-living and particle-associated size fractions. In both size fractions, distribution patterns of bacterial taxa, and species within taxa, displayed temporal and spatial preferences. Distinct bacterial assemblages specific to season and depth in the water column were identified. These distinct assemblages, consistent for both 2014 and 2016, suggested replicable patterns in microbial communities for spring and fall in this region. Over all sites, temperature and oxygen values were highly correlated with community similarity, and salinity and oxygen values were the most strongly positively- and negatively correlated with alpha diversity, respectively. However, the strengths of these correlations depended on the depth and season sampled. The bathymetry of the Scotian Shelf, the abrupt shelf break to the Scotian Slope and the major ocean currents dominating in the region led to the formation of distinct on-shelf and off-shelf bacterial communities both in spring and fall. The highest species richness was observed at the shelf break, where water masses from the two major currents meet. Our study establishes the baseline for assessing future changes in the bacterial community of the Scotian Shelf waters, a rapidly changing sector of the Atlantic Ocean.

## Introduction

Microbes are the main drivers of biogeochemical cycles in the ocean (Falkowski et al., 1998). Given their essential roles in regulating global nutrient cycles and primary productivity (Arrigo, 2005), understanding the factors that shape microbial community structure on spatial and temporal scales is crucial for predicting the effects of climate change on marine ecosystems (Hanson et al., 2012). It is now well established that patterns in marine microbial community structure are observable across latitude, longitude and depth, as well as temporally (Zinger et al., 2011). The processes shaping the patterns in microbial biogeography in the global ocean have been the subject of debate, with most of the controversy revolving around whether historical processes or contemporary selection has the greatest effect on the observed distribution patterns (Hewson et al., 2006; Schauer et al., 2010; Ghiglione et al., 2012; Sul et al., 2012; Monier et al., 2014; Zinger et al., 2014; Nguyen and Landfald, 2015). By definition, contemporary selection refers to the ability of a given microbial species to thrive in a set of local environmental and biological conditions (Lima-mendez et al., 2015; Milici et al., 2016a; Needham and Fuhrman, 2016), while historical processes refer primarily to rates of dispersal and the effect of past environmental conditions on the genetic composition of the microbial community (Martiny et al., 2006). One leading theory on microbial biogeography, coined by Baas-Becking decades ago: “Everything is everywhere, but the environment selects” (Baas Becking, 1934), is based on the premise that small size, extremely large populations and high turnover rates of microbes make dispersal limitation negligible, instead attributing differences in microbial species distribution to contemporary selection driven by local environmental factors (Green and Bohannan, 2006; Hanson et al., 2012). A limited number of studies have attempted to address the roles of these multiple processes in shaping biogeographical patterns observed in microbial communities (Rusch et al., 2007; Fortunato et al., 2012, 2013; Ghiglione et al., 2012 Gilbert et al., 2012), at times reaching opposite conclusions (Ghiglione et al., 2012; Sul et al., 2012). While local environmental factors are characterized by measuring a suite of physical and chemical variables, the role of dispersal is mostly assessed from the geographical distance between communities (Lindström and Langenheder, 2012), a metric that ignores the connectivity or isolation of water bodies created by oceanographic features such as currents, oceanic fronts, and eddies (Hernando-Morales et al., 2017). An increasing number of studies have demonstrated that hydrographic features play an important role in controlling the microbial community structure at the regional level (Baltar et al., 2016; Dinasquet et al., 2017; Hernando-Morales et al., 2017; Venkatachalam et al., 2017; Raes et al., 2018). Taking these new findings into consideration, we use here an extensive dataset collected on the Scotian Shelf (SS) region of the Northwest Atlantic Ocean (NA) to describe the microbial community in spring and fall, and assess the role of environmental variables and of known circulation patterns in shaping the observed microbial community structure.

Extending offshore between 120 and 240 km from Nova Scotia, Canada, the SS is a region of complex bathymetry characterized by a series of deep basins (up to almost 300 m) and shallow (<100 m) offshore banks. Beyond the shelf break, water depths drop off rapidly to >3000 m. Ocean conditions in the region are influenced by the predominantly equatorward northwest Atlantic shelf currents which bring cold, low salinity water to the region, while the warm water influence of the Gulf Stream, which flows to the northeast offshore of the SS, is observed in the slope waters and on the western shelf in the fall (Loder et al., 1998; Hannah et al., 2001). These two main currents result in a general pattern of increasing temperature and salinity across the SS from the NE down to the SW (Drinkwater and Gilbert, 2004). In addition, warmer and more saline offshore waters beyond the shelf slope result in a gradient of increasing salinity and temperature from the inshore region to the offshore region (Drinkwater and Gilbert, 2004; Dasilva et al., 2014). The mean annual surface temperature on the SS has risen 0.80°C over a 50 years period and the number of extreme heat events has increased significantly in the last 30 years (Hebert et al., 2018). The seasonal warming is also occurring earlier in the year in this region, compounding to trends in warmer water (Wethey and Lima, 2012). Although there are reports that biogeographic ranges of some invertebrate species found in this region are contracting toward the poles (Jones et al., 2010), the impact of these warming trends on microbial communities is currently undetermined.

Twice yearly since 1999, the Atlantic Zone Monitoring Program (AZMP) implemented by Fisheries and Oceans Canada, has collected extensive physical, chemical and biological observations on the SS (Therriault et al., 1998). To date, studies of microbial communities on the SS have focused primarily on phytoplankton communities characterized by flow cytometry, microscopy and sequencing of the eukaryotic SSU rRNA genes (18S rRNA) (Li, 2002; Li et al., 2006, 2011; Dasilva et al., 2014; Luddington et al., 2016). In contrast, characterization of bacterial communities have been limited geographically and in scope to two sites on the SS; the Thebaud platform, a site of oil exploration, and the Gully, a submarine canyon at the edge of the continental shelf in Nova Scotia (Yeung et al., 2010, 2011). Thus, a comprehensive overview of the bacterial community structure of the region and its relationship to environmental variability is lacking.

In this study, the bacterial community of the SS was characterized using high-throughput sequencing of 16S rRNA gene amplicons for spring and fall missions in 2014 and 2016. A detailed set of samples collected in 2014 was used to explore the environmental drivers of bacterial diversity and community structure at a regional scale along the SS. In particular, we tested whether temperature and species richness were positively correlated, because temperature has previously been identified as one of the most important environmental factors spatially shaping the structure of microbial communities in the epipelagic sunlit oceanic surface waters (Sunagawa et al., 2015). The North Atlantic Ocean is changing rapidly in response to climate change (Wethey and Lima, 2012; Saba et al., 2015). Our study provides the first extensive characterization of the bacterial community in this economically and environmentally important marine sector. The rapidly changing temperature distribution and the expected changes predicted for this region of the North Atlantic warrant the establishment of a baseline microbial community structure, albeit a shifting one, for future comparisons.

## Experimental Procedures

### Sample Collection

**FIGURE 1:**
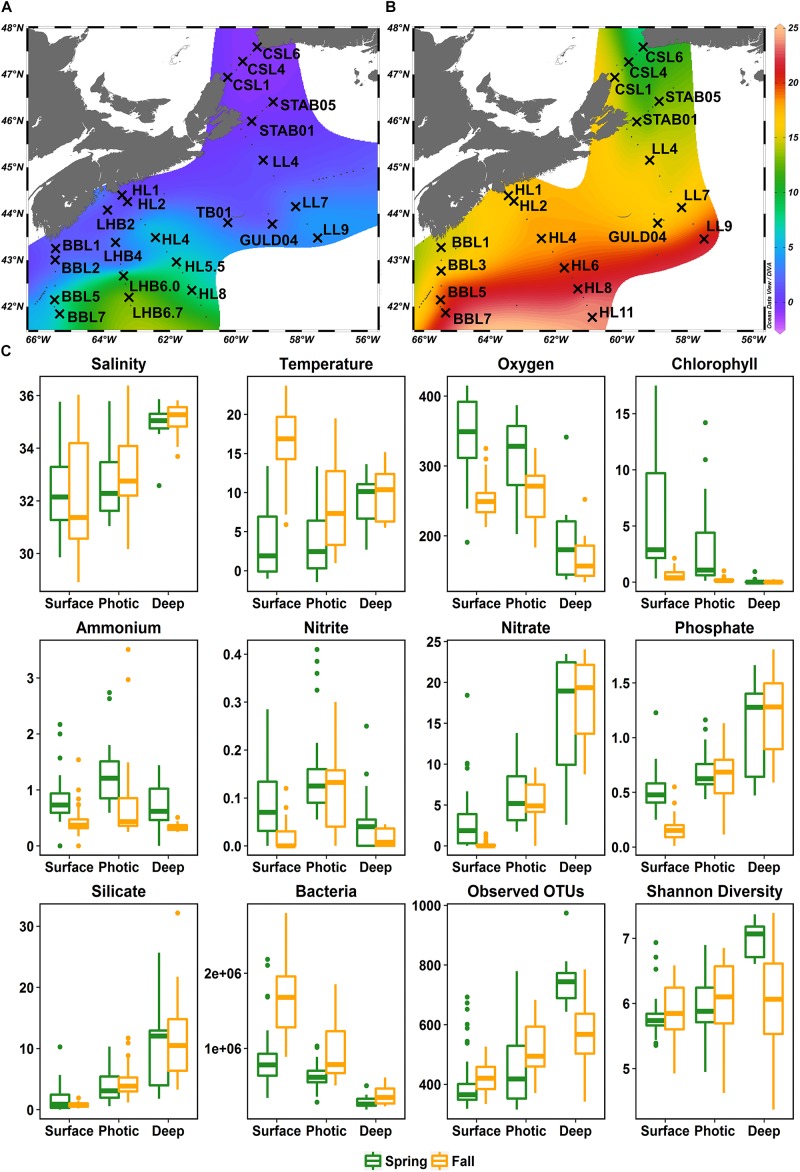
Map of Scotian Shelf stations sampled aboard the *CCGS Hudson* in April **(A)** and September/October **(B)** of 2014, overlaid on surface temperatures during the time of sampling. Stations sampled are labeled with crosses and the transect acronym followed by the station number. Transects: BBL, Browns Bank Line; LHB, LaHave Basin Line; HL, Halifax Line; TB, Thebaud Platform Station; GULD, Gully Station; LL, Louisbourg Line; STAB, St. Anns Bank Line; CSL, Cabot Strait Line. **(C)** Boxplots showing the ranges of environmental variables and free-living bacterial diversity across the shelf split into three depth groups and the two seasons sampled. Salinity (PSU), temperature (°C), oxygen (μmol/kg), chlorophyll *a* (mg/m^3^), ammonium (mmol/m^3^), nitrite (mmol/m^3^), nitrate (mmol/m^3^), phosphate (mmol/m^3^), silicate (mmol/m^3^), bacteria (cells/mL), number of OTUs, Shannon diversity index.

Water samples were collected in 2014 on the spring and fall AZMP missions aboard the *CCGS Hudson* (HUD2014004 April 4-23, HUD2014030 September 19 – October 12) at stations throughout the SS and Scotian Slope, occasionally reaching Gulf Stream influenced off-shelf waters (Figure 1). Six sections including the Browns Bank Line (BBL), the LaHave Basin Line (LHB), the Halifax Line (HL), the Louisbourg Line (LL), the St. Anns Bank Line (STAB), and the Cabot Strait Line (CSL) were extensively surveyed in 2014. In addition, during the spring leg, the station GULD04 corresponding to the ecologically rich shelf break Gully region, and the Thebaud platform (TB01) station located in close proximity to the Thebaud oil and gas platform were sampled (Figure 1A). Variations in cruise tracks led to slight differences in stations sampled in the fall and spring cruises. In total, 42 stations were sampled at 4 depths, resulting in 168 samples (92 samples from spring and 76 from fall cruises) with 64 sampling sites shared between the two 2014 cruises (Figure 1A,B).

We compared the microbial community similarity at stations along the Halifax line, a section sampled in the spring and fall AZMP cruises of 2014 and 2016. Repeat sampling of HL was carried out in 2016 AZMP missions (HUD2016003 April 9–25, HUD2016027 September 25 – October 6), resulting in an additional 65 samples for comparison with 2014 (Supplementary Table S1). At each selected station, 4 L of water was collected from each of 4 depths and pre-filtered through a 160 μm (2014) or a 330 μm (2016) mesh to remove mesozooplankton. The water was then filtered through a 3 μm polycarbonate Isopore filter (Millipore, United States) to capture particle associated (PA) bacteria and then redistributed into 4 L bottles and filtered using a vacuum (2014), or a peristaltic pump (2016) through 0.2 μm polycarbonate Isopore filters (Millipore, United States) to capture free-living (FL) bacteria. Both the 3 and 0.2 μm filters were stored at -80°C. At all stations sampled, 1 and 20 m water samples were collected, while the other two depths were selected based on the depth of the ocean floor and oceanographic features in the vertical depth profile (Supplementary Table S2). Samples were also collected from the water column oxygen minima when present, usually corresponding to a depth of 250 ± 50 m. Bacterial abundance was measured using flow cytometry. Parallel 1.8 mL seawater samples for analytical flow cytometry (AFC) were fixed with 1% paraformaldehyde (Alfa-Aesar, United States), incubated at room temperature for an hour, then stored at -80°C for later analysis. Nutrient and chlorophyll measurements were made using standard AZMP protocols (Mitchell et al., 2002).

Samples were categorized based on season (spring or fall), and depth: Surface (1–20 m), Photic (40–80 m), and Deep (100–300 m). The discrete depth categories were based on the average photic zone depth from over 10 years of observations by the Bedford Institute of Oceanography (BIO) on the SS (Johnson et al., 2014). Specifically, all surface and deep samples were consistently taken within the photic zone and aphotic zone, respectively. Samples collected at the intermediate depths in the photic zone were within the range of photic depth variability throughout the year, thus the amount of light these samples received was variable and lower than surface depths. A subset of samples from cross-shelf sections (BBL, LHB, HL, STAB1-LL; Figure 1 and Supplementary Figure S1) were used to investigate the communities at the shelf-break. These samples were grouped into categories on-shelf (BBL1, 2, 3; LHB2, 4; HL1, 2; STAB1; LL4), shelf break (BBL5; LHB6; HL4, 5.5, 6; LL7), and off-shelf (BBL7; LHB6.7; HL8, 11; LL9) based on geographic position relative to the shelf break and spatial patterns of surface temperature and salinity (Supplementary Figures S1, S2A,B,F,G). Temperature-salinity diagrams for all transects by season (not shown) were also used to confirm that the on-shelf and off-shelf groupings reflected distinct water masses of the Scotian shelf (SS).

### DNA Extraction, Library Preparation and Illumina MiSeq Sequencing

DNA was extracted from technical duplicate 0.2 and 3 μm polycarbonate filters using the DNeasy Plant Mini Kit (Qiagen, Germany) according to the manufacturer’s instructions with some minor modifications in the cell lysis procedure. Fifty microliters of lysozyme (5 mg/mL) (Fisher BioReagents, United Kingdom) was initially added to each filter and each sample was vortexed on high for 30 s. Then 400 μL of lysis buffer AP1 (from the DNeasy Plant Mini Kit) was added to each sample tube followed by the addition of 45 μL of proteinase K (20 mg/mL) (Fisher BioReagents, United Kingdom). The samples were then incubated at 55°C with shaking for 1 h. After this incubation, 4 μL of RNase A (Qiagen, Germany) was added to the samples, which were then kept on ice for 10 min. From this point on, the extraction followed the manufacturer’s protocol, with a final elution of the DNA in 100 μL of elution buffer. DNA concentrations and purity were measured with a NanoDrop 2000 (Thermo Scientific, United States).

The samples were prepared for sequencing on an Illumina MiSeq instrument, following the Microbiome Amplicon Sequencing Workflow (Comeau et al., 2017). Each DNA sample was amplified using dual-indexing Illumina fusion primers that targeted the V6–V8 438 bp region of the bacterial 16S rRNA gene (Comeau et al., 2011). We used the forward B969F (ACGCGHNRAACCTTACC), and the reverse BA1406R (ACGGGCRGTGWGTRCAA) primer. The full primer sequences, including fusion sequences and adapters are listed in Supplementary Table S3. Each DNA sample was amplified as an undiluted template and at a 1:10 template dilution, to reduce the potential effects of PCR bias. The PCR products from diluted and undiluted templates were pooled and their quality was verified using an E-gel 96-well high-throughput system (Invitrogen, United States). Library normalization and PCR clean-up was conducted using a SequalPrep 96-well Plate Kit (Invitrogen, United States). After normalization, all samples were pooled together, and the final library pool was quantified using a Qubit with PicoGreen (Invitrogen, United States). Finally, the pooled samples were run on an Illumina MiSeq sequencer using paired-end 300 + 300 bp v3 chemistry. The MiSeq on-board software demultiplexed the reads, creating one forward and one reverse read file per sample. Raw sequence files are available at the NCBI Sequence Read Archive under accession SRP076591, and PRJNA325151.

### Bacterial Abundance

Analytical flow cytometry (AFC) was used to characterize bacterial abundance. Samples were analyzed with a BD Accuri Flow Cytometer (BD Biosciences, United States). Measurements of bacterial concentrations were made by adding SYBR (Invitrogen, United States) stain to the sample and incubated in the dark for 15 min. The SYBR stained samples were run with a threshold of 800 at FL1 and the gates used to determine bacterial counts followed a bacterial gating strategy developed for the Accuri instrument (Gatza et al., 2013; Prest et al., 2013).

### QIIME 16S rRNA Data Analysis

Preliminary analysis and processing of 16S rRNA gene sequences followed a QIIME version 1.8.0 (Caporaso et al., 2010) pipeline workflow (Comeau et al., 2017). The program PEAR version 0.9.6 was first used to merge the demultiplexed, paired-end sequences together (Zhang et al., 2014). After merging paired ends, sequences less than 400 bp in length or with a quality less than 30 over 90% of bases were discarded. Chimeric sequences were removed using UCHIME (Edgar et al., 2011). Operational Taxonomic Units (OTUs) were clustered based on 97% sequence similarity using *sortmerna* (Kopylova et al., 2012) for reference picking and *sumaclust* (Mercier et al., 2013) for *de novo* OTU picking (i.e., “open-reference” picking). This process used the reference Greengenes database version 13.8 (McDonald et al., 2012) for preliminary OTU picking, and then subsampled failed sequences using *de novo* picking. OTUs that were identified by less than 0.1% of reads were removed to account for bleed-through between runs on the Illumina MiSeq (Comeau et al., 2017), and the remaining OTUs were used for further analysis. In order to focus the study on the bacterial community, additional quality control measures included removing all sequences assigned to mitochondria, and chloroplasts, as well as the few reads that were assigned to Archaea, because the latter is poorly represented in sequence reads obtained from the V6-V8 variable region, in general. The Greengenes database used above, erroneously classified the family SAR86 within the class of Gammaproteobacteria as the genus “*Candidatus Portiera*”, which is a known endosymbiont of the white fly *Bemisia tabaci* (Jiang Z.F. et al., 2012). All OTUs assigned to the *Candidatus Portiera* classification were reclassified as belonging to the family of SAR86. A BLAST search of these misclassified sequences supported this decision. Analysis of the microbial community structure was conducted on the 9364 remaining OTUs that passed the quality control steps listed above. For indicator species analysis, only 390 OTUs that reached a relative abundance greater than 1% in at least one sample across the SS were included to avoid spurious observations.

To compare the relative abundance of OTUs between samples collected in 2014, sequence reads were rarefied to a sequencing depth of 7500 which corresponded to the duplicate sample pair with the lowest combined sequencing depth. The observations from the remaining duplicate samples were combined by averaging using the QIIME script *collapse_samples.py*. The comparison of the 2014 and 2016 samples from the Halifax line (HL) was conducted in a separate workflow; the samples were combined and rarefied to a sequencing depth of 5000 for downstream statistical analysis.

### Statistical Analysis and Data Visualization

Statistical analyses were conducted using either base R version 3.2.1 (R Core Team, 2016) or the specific R packages described below. Figures, excluding maps, were made using *gplots* (Warnes et al., 2016) or *ggplot2* (Wickham and Chang, 2015) packages in R. CTD data was extracted in R using the package *oce* (Kelley et al., 2018). Ocean Data View version 4.6.5 (Schlitzer, 2015) was used to create images featuring surface maps and transects of the SS. Density shading using the DIVA (Data-Interpolating Variational Analysis) gridding algorithm was implemented through Ocean Data View to visualize approximate spatial distribution of environmental variables and the distribution of select taxa (Barth et al., 2010).

#### Measures of Alpha and Beta Diversity

The Shannon diversity and Chao1 richness (Chao, 1984) indices, measures of alpha diversity, were calculated using the rarefied dataset through the QIIME script *alpha_diversity.py*. Non-Metric Multidimensional Scaling (NMDS) plots were created using the *metaMDS* function from the R package *vegan* (Dixon, 2003; Oksanen et al., 2017). The abundance data was Hellinger transformed prior to conducting the NMDS analysis on Bray–Curtis dissimilarities. The Hellinger transformation is recommended for use on species abundance data and gives low weights to variables with low counts and many zeroes (Legendre and Gallagher, 2001; Ramette, 2007; Buttigieg and Ramette, 2014). Analysis of similarities (ANOSIM) tests were performed to test for significant differences in the community assemblages of various groups such as depth, season, year, and size fraction. The ANOSIM tests were performed on the same data used for generation of the NMDS plots, using the function *anosim*, from the R package *vegan*.

To address the variability between the 2014 and 2016 sampling of the HL section, pairwise comparisons of Bray–Curtis dissimilarity between samples were used. For the analysis of biogeographical patterns, partial Mantel tests were performed on distance matrices of the dissimilarities in bacterial composition, environmental parameters, and geographic position between samples. The analyses were carried out for the complete data of each size fraction, on spring and fall data from each size fraction, and data divided by depth from each size fraction to identify overall, seasonal, and depth-related drivers of community structure, respectively. For the abundance matrices, Bray–Curtis dissimilarities were calculated after Hellinger transformation. The variables included in the environmental distance matrix were temperature, salinity, oxygen, nitrite, nitrate, and ammonium (all non-collinear (*r* < 0.7) environmental variables). All environmental variables were standardized (centered and scaled) before the Euclidean distance between sites was calculated. Partial Mantel Tests were then calculated with the *mantel.partial* function from the package *vegan* (Spearman’s rank correlation, 10000 permutations) in order to test the significance of the correlation between either geographic distance or environmental distance, and community similarity while controlling for the influence of the other matrix. Geographic distance between sites was calculated as Haversine distance with the package *geosphere* (Hijmans, 2016) in R. An additional test comparing the Bray–Curtis community similarity of the FL and PA size fractions was conducted for all subsets of the data.

To further determine which specific environmental variables best explained the variations in community structure across the SS, Partial Mantel Tests were employed as described above, but with a single environmental variable contributing to the environmental matrix. The geographic distance matrix was again utilized as the control for these tests. In this way, the effect of each individual environmental variable on community structure could be determined over all sites or compared across seasons and depth. To test for differences in the communities between the on-shelf, shelf break, and off-shelf zones from the across shelf transects, Permutational Multivariate Analysis of Variance (PERMANOVA) was conducted using the function adonis from the package vegan (Oksanen et al., 2017) using the Bray–Curtis dissimilarity with 1000 permutations. Pairwise *post hoc* tests were conducted using the function pairwise.adonis from package pairwiseAdonis (Martinez Arbizu, 2017) with Bonferroni correction.

#### Indicator Species

Microbial indicator species are defined here as a single species or a small group of species that represent a specific environment or biological association such as a specific community. Indicator species may be used to detect environmental changes in a geographic area or by association predict the diversity and composition of a specific microbial community within an area. Here we identified indicator species for each season, region and size fraction using the *multipatt* function from the *indicspecies* package in R, using the point-biserial correlation index (De Cáceres et al., 2010).

## Results

### The Scotian Shelf (SS) Oceanographic Conditions in Spring and Fall

Temperature and salinity increased with increasing distance from shore in both spring and fall (Figure 1A,B, Supplementary Figure S2, and Supplementary Table S2). Surface and photic zone temperatures were much warmer during the fall (surface average: 16.5°C, photic average: 8.4°C) than during the spring cruise (surface average: 3.3°C, photic average: 3.9°C), while temperatures at depth generally fell within the same range (5–15°C) in both seasons (Figure 1C). During both spring and fall, dissolved O_2_ concentrations were higher in the surface and photic zones than at depth (Figure 1C). The average O_2_ concentration in the fall was lower (241 μmol O_2_/kg) than in the spring (313 μmol O_2_/kg) (Figure 1C). Concentrations of ammonium, nitrate, nitrite and phosphate, in surface waters were generally higher in the spring than in the fall (Figure 1C). Nitrate, phosphate, and silicate concentrations increased dramatically with depth in both seasons, but ammonium and nitrite were relatively constant throughout the water column (Figure 1C). The average chlorophyll *a* concentrations were significantly higher (*W* = 5717, *p-*value < 0.001, Mann–Whitney-*U* Test) in the spring (maximum of 17.49 mg/m^3^ at STAB_01 [20 m]; average 3.97 mg/m^3^) than in the fall (maximum of 2.13 mg/m^3^ at CSL_01 [1 m]; average 0.39 mg/m^3^).

**FIGURE 2:**
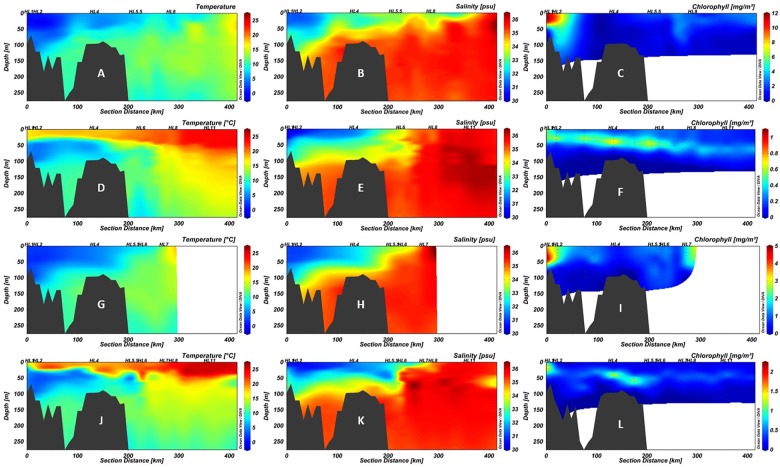
Section plots of the Halifax Line (HL) in 2014 spring **(A–C)**, 2014 fall **(D–F)**, 2016 spring **(G–I)**, and 2016 fall **(J–L)**. The first column **(A,D,G,J)** shows temperature (°C), the second **(B,E,H,K)** salinity (psu), and the third **(C,F,I,L)** chlorophyll *a* (mg/m^3^). Note that while color scales for temperature and salinity are consistent between panels, those for chlorophyll *a* differ due to high variability between cruises. Points indicate locations of data collection; black polygons are bottom contour (GEBCO 2014 6 × 6 min). Stations sampled in this study are labeled above the plots. The 200 km point represents the average location of the Shelf-Slope Front (SSF).

The physicochemical characteristics of the water column of HL in 2016 were comparable to that observed between spring and fall of 2014, reflecting the SS seasonal patterns (Figure 2) with respect to temperature, salinity, and chlorophyll concentrations (Figure 2). Surface waters exceeded 20°C throughout the section in fall of both years (Figure 2D,J), while lower water salinity down to approximately 75 m was observed on the shelf as far as the shelf break (Figure 2E,K). Although qualitatively similar, the absolute magnitude of chlorophyll *a* concentrations was much higher in spring of 2014 than in 2016 (Figure 2C,I). Both surface and deep water temperatures were higher than average in 2014, compared to the period 1981–2010, with the strongest positive anomalies in surface waters in the second half of the year (DFO, 2015; Hebert et al., 2015). Variation in seasonal temperature may cause slight difference in the timing of the onset of the spring bloom relative to the AZMP cruise dates in 2014 and 2016 (DFO, 2017).

### Scotian Shelf Microbial Community Composition

Bacterial concentrations decreased steadily with depth but were significantly higher in the fall than in the spring, with an average of 1.3 × 10^6^ cells/mL and 0.7 × 10^6^ cells/mL, respectively (*W* = 5286, *p-*value < 0.001, Mann–Whitney-*U* Test) (Figure 1C). For all samples combined, 9364 OTUs from the FL and PA size fractions were identified representing 35 phyla (Figure 1C).

#### Comparison of Spring and Fall Microbial Communities in 2014 and 2016

**FIGURE 3:**
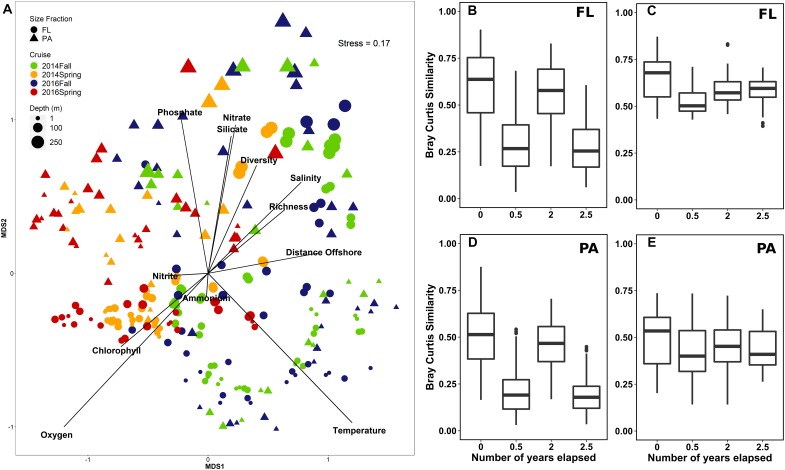
Comparison of Halifax Line (HL) spring and fall communities in 2014 and 2016 (*n* = 252) in the free-living (FL) and particle-attached (PA) size fractions. **(A)** Non-metric multidimensional scaling analysis of Hellinger transformed proportional OTU abundance data using the Bray-Curtis dissimilarity matrix. The symbols for the samples are colored based on season, their shape represents the different size fraction, and the size of the symbol indicates the sampling depth in the water column, on a continuous scale from 1-200 m. Environmental vectors were fit onto the ordination using the function *envfit*. Diversity refers to the Shannon diversity index. **(B–E)** Boxplots showing the range of pairwise comparisons of Bray-Curtis similarity between samples separated by the same number of seasons from the surface **(B,D)** and deep **(C,E)** waters.

A comparison of the bacterial community for HL in 2014 and 2016 showed that for the communities in both years, the shallow microbial communities segregated according to the season, with spring communities from both 2014 and 2016 more similar to each other than with their respective annual fall counterparts (Figure 3A). However, pairwise comparisons indicated that the interannual variation was still statistically significant for both size fractions, with a very low difference of mean ranks (FL: *R* = 0.05, *p* < 0.01; PA: *R* = 0.06, *p* < 0.05; ANOSIM test for seasonal differences between 2014 and 2016) (Figure 3). Pairwise comparisons of Bray–Curtis dissimilarity between surface samples separated by year and season, indicated that samples collected in the same season of the same year showed the highest similarity, followed by samples collected 2 years apart (i.e., same season, different years), and finally by those collected in different seasons (spring of 2014 and fall of 2016; Figure 3B,D). The difference in community composition between spring and fall was reduced with depth as can be seen in the NMDS plot (Figure 3A). However, deep samples were still observed to have significant differences in Shannon diversity in different seasons [Kruskal–Wallis: chi-squared (1) = 5.427, *p* = 0.020] and Chao1 richness between years [Kruskal–Wallis: chi-squared (1) = 4.550, *p* = 0.033].

#### Microbial Community Composition of the Scotian Shelf in 2014

**FIGURE 4:**
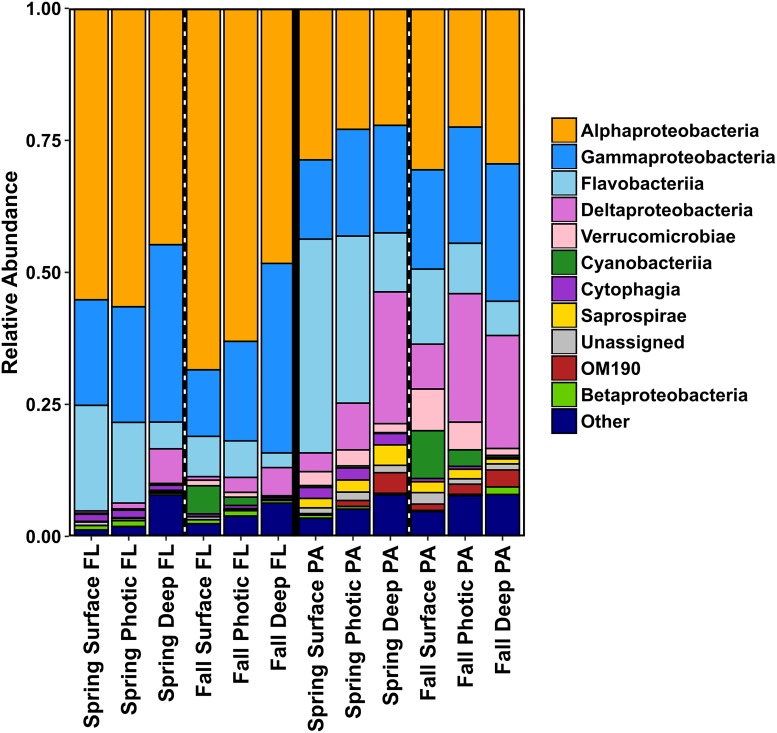
Bar charts showing relative abundances of the bacterial community taxa of the different size fractions (PA and FL), split into season and depth categories for 2014. Results are summed and averaged for all samples within each season and depth category.

The bacterial community of the SS was highly diverse and the relative abundance of taxa varied with season, depth, and size fraction. Alphaproteobacteria and Gammaproteobacteria were the dominant taxa in the FL category, in both spring and fall samples and all depths (Figure 4 and Supplementary Figure S3). The relative abundance of Flavobacteriia and Deltaproteobacteria was almost as high as the Alpha- and Gammaproteobacteria in the PA samples, and Synechococcophycideae taxa were present mainly in the fall surface waters in both the FL and PA fraction. The PA size fraction was enriched in select bacterial taxa within Flavobacteriia, Deltaproteobacteria, Verrucomicrobiae, Saprospirae, and various classes of Planctomycetes (Figure 4). The candidate phylum OM190 (Planctomycetes) was found mainly in the PA fraction and had higher relative abundance in the deep water samples. OTUs belonging to the Saprospiraceae were also found predominantly in the deep water samples of the PA fraction in the spring (Figure 4).

**FIGURE 5:**
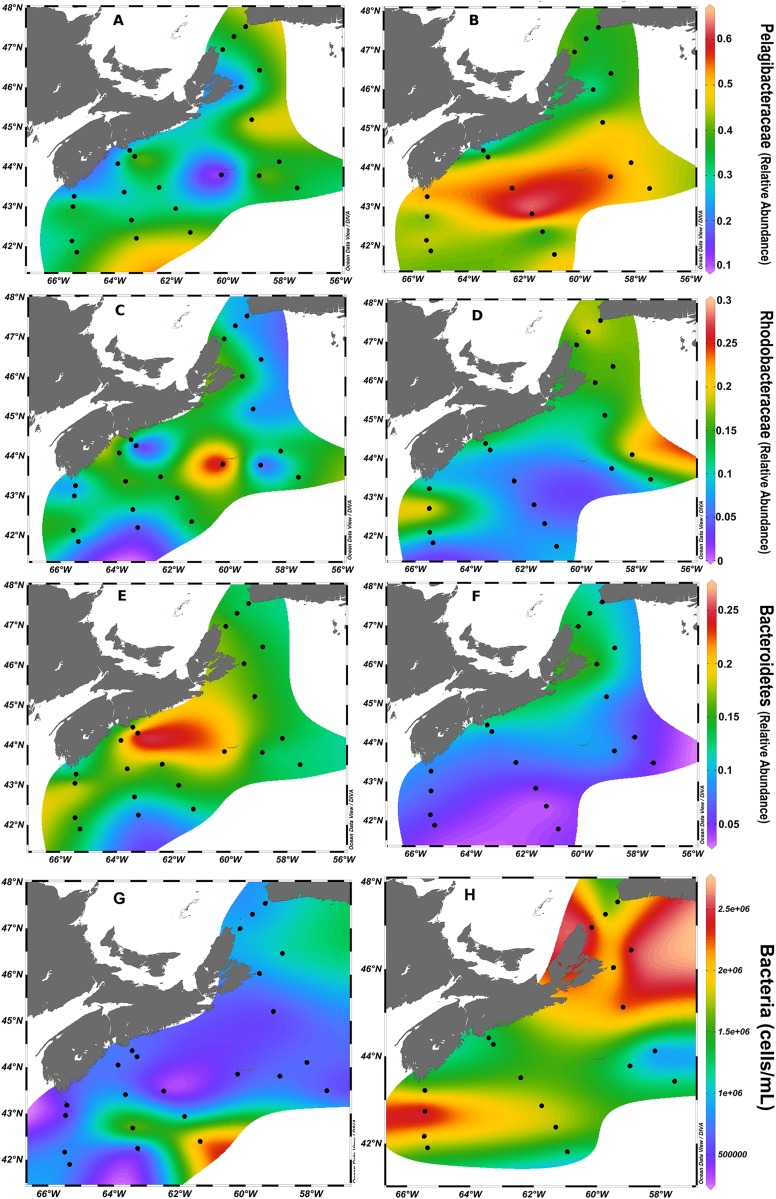
Surface plots, from the 1 m depth, of taxa over the SS in spring **(A,C,E,G)** and fall **(B,D,F,H)**. Pelagibacteraceae **(A,B)**, Rhodobacteraceae **(C,D)**, and Bacteroidetes **(E,F)** groups are displayed. Note that color scales are fixed between seasons of the same taxa, but vary between taxa. Black dots mark surface sites. **(G,H)** Represent the bacterial cell density in spring and fall, respectively. Bacterial cell density was overall correlated with temperature and was higher in the fall (Supplementary Figure S4).

Within the FL fraction, members of the Pelagibacteraceae and of the Rhodobacteraceae were dominant families within Alphaproteobacteria that were recovered in high relative abundance throughout the SS waters in both seasons (Supplementary Table S4). Pelagibacteraceae accounted on average for 35% of the relative abundance over all sites. The dominance of this taxon at individual sites varied across the SS, with a maximum relative abundance of Pelagibacteraceae of 67% at station HL4 (40 m, fall) (Supplementary Figure S3). Rhodobacteraceae and Bacteroidetes, the next most represented taxa in the FL fraction, also exhibited wide ranges in relative abundance across sites, averaging 13% for Rhodobacteraceae (up to 48% at LL7 [250 m, fall]) and 11% for Bacteroidetes (up to 27% in spring) (Figure 4 and Supplementary Figure S3). We observed that the relative abundance of several taxa varied spatially and between sampling seasons on the SS (Figure 5A–F). The relative abundance of Pelagibacteraceae was higher in the fall (Figure 5A,B), while Bacteroidetes was higher in spring (Figure 5E,F). In spring, the distributions of Rhodobacteraceae and Pelagibacteraceae mirrored each other (Figure 5A,C), while Rhodobacteraceae dominance in the bacterial community shifted to the northeast (Figure 5C,D) in the fall. As expected, many OTUs belonging to Pelagibacteraceae were indicator species for the FL fraction. In particular, Pelagibacteraceae sp. OTU#637092, with the highest relative abundance overall in the FL fraction, was also the most significant indicator species for this fraction, followed by OTUs belonging to Oceanospirillales, Rhodobacteraceae, and other Pelagibacteraceae (Supplementary Figure S5 and Supplementary Table S5). Evidence for differing niche preferences of OTUs within the same broad taxonomic groups was most pronounced for the numerous OTUs belonging to Pelagibacteraceae, which dominated the FL fraction due to their small cell size, but were otherwise selectively distributed throughout all habitats sampled in this study. For instance, 16 of the 34 OTUs from Pelagibacteraceae were identified as indicator species for the fall, while 8 were identified as indicator species for the spring season (Supplementary Table S5). A complete list of representative Pelagibacteraceae and Rhodobacteraceae indicator OTUs for depth, season and size fractions can be found in Supplementary Table S5.

**FIGURE 6:**
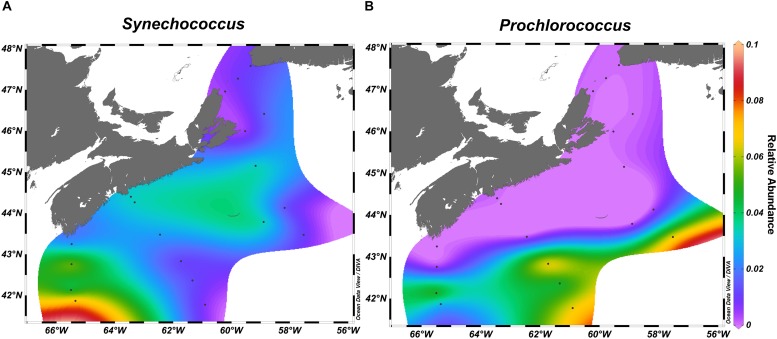
*Synechococcus* **(A)** and *Prochlorococcus* **(B)** relative abundance across the surface of the Scotian Shelf in fall.

We observed zonation in the microbial communities with OTUs belonging to Acidobacteria, Deltaproteobacteria, SAR406, and Planctomycetes more often recovered in the deep water samples, while Cyanobacteria and Flavobacteriia were found in the surface ocean. The strongest indicator species for the deep water samples included several Pelagibacteraceae OTUs (Supplementary Tables S5, S6). Top indicator species for the surface waters were OTUs assigned to diverse Flavobacteriaceae, Alphaproteobacteria sp. Rhodobacteraceae sp., and *Synechococcus* sp. (Figure 6, Supplementary Figure S5 and Supplementary Tables S5, S6).

### Environmental Factors Driving Bacterial Diversity on the Scotian Shelf

Bacterial cell density was significantly linearly correlated with temperature (adjusted *R*^2^ = 0.26, *F*_1,122_ = 44.14, *p* < 0.001) in a regression analysis that included all spring and fall samples, albeit with a stronger contribution from the fall samples due to a larger temperature range (Supplementary Figure S4) and overall higher cell density during that season (Figure 1C, 5G,H). The following results report on the composition and diversity of the bacterial community, independently of absolute abundance of individual taxa. We observed that the alpha diversity, measured both with number of distinct OTUs or Shannon diversity index, was positively correlated between the FL and PA fractions (Supplementary Figure S6) and overall higher in the PA fraction. Statistically significant correlation between the FL and PA fractions persisted in samples from specific depths (Surface: Adjusted *R*^2^ = 0.3814, *p*-value < 0.0001; Photic: Adjusted *R*^2^ = 0.1735, *p*-value < 0.01; Deep: Adjusted *R*^2^ = 0.4675, *p*-value < 0.0001). Thus, samples exhibiting high diversity in the PA size fraction were also likely to exhibit high diversity in the FL size fraction. This relationship was stronger for species richness (observed OTUs, Supplementary Figure S6A) compared to the Shannon diversity index that takes into account both species richness and evenness (Supplementary Figure S6B). The Shannon diversity index was higher in the PA size fraction than in the FL size fraction, regardless of season (both seasons: *W* = 21398, *p* < 0.001; fall: *W* = 1208, *p* < 0.001, spring: *W* = 1352, *p* < 0.001; Mann–Whitney-*U* Test).

**FIGURE 7:**
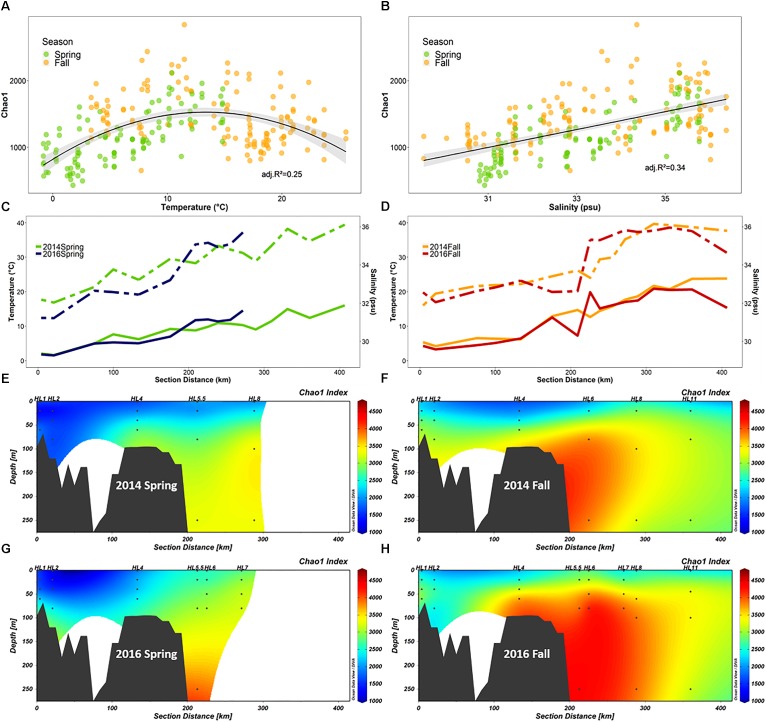
Diversity of the microbial community (FL and PA combined) along the Halifax Line (HL) section in spring and fall of 2014 and 2016 (*n* = 252). **(A,B)** Show Chao1 richness index as a function of salinity and temperature, respectively. The maximum Chao1 value was observed at 13.3°C. **(C,D)** Show line graphs of salinity (dashed) and temperature (solid) at 50 m depth throughout the HL transect in spring and fall, respectively. **(E–H)** Show the section plots of Chao1 index (summed over FL and PA samples) in both 2014 and 2016 spring (**E,G**, respectively) and fall (**F,H**, respectively) cruises. Points indicate locations of data collection; gray polygons are the bottom contour (GEBCO 2014 6 × 6 min). Stations sampled in this study are labeled above the plots.

Several environmental factors were directly correlated to bacterial community diversity (Table 1). Overall, the Shannon diversity index and species richness (Chao1 index) were significantly positively correlated with salinity and other factors such as temperature and nutrients, while oxygen, chlorophyll *a* and latitude showed a negative correlation (Table 1; *p* < 0.05; Figure 7A,B). Although the strength of the correlation with diverse environmental variables varied with season, and depth, the sign of the statistically significant correlations remained the same across all categories with salinity, nutrients, depth and temperature showing a positive correlation with diversity, while oxygen, chlorophyll a, latitude and bacterial abundances were negatively correlated with diversity (Table 1).

**Table 1:** Spearman correlations between bacterial diversity (Shannon index) of the free-living (FL) size fraction and environmental variables with significant values indicated in bold.

	All Sites	Spring				Fall			
		Surface	Photic	Deep	All	Surface	Photic	Deep	All
Salinity	0.56^∗∗∗^	0.24	**0.77^∗∗∗^**	–0.22	0.63^∗∗∗^	**0.50^∗∗∗^**	**0.70^∗∗∗^**	–0.51	**0.47^∗∗∗^**
Silicate	0.42^∗∗∗^	**0.47^∗∗∗^**	0.36	**0.64^∗^**	0.60^∗∗∗^	**0.34^∗^**	–0.12	0.33	**0.23^∗^**
Nitrate	0.41^∗∗∗^	**0.56^∗∗∗^**	**0.71^∗∗∗^**	0.48	0.72^∗∗∗^	–0.24	0.18	0.02	0.12
Depth	0.41^∗∗∗^				0.56^∗∗∗^				0.20
Temperature	0.29^∗∗∗^	0.19	**0.74^∗∗∗^**	–0.29	0.57^∗∗∗^	**0.59^∗∗∗^**	**0.46^∗^**	–0.51	0.19
Phosphate	0.24^∗∗^	**0.42^∗∗^**	0.19	0.47	0.52^∗∗∗^	**–0.43^∗∗^**	–0.29	0.23	–0.01
Oxygen	–0.53^∗∗∗^	**–0.32^∗^**	**–0.80^∗∗∗^**	–0.43	–0.68^∗∗∗^	**–0.62^∗∗∗^**	**–0.61^∗∗^**	0.33	**–0.42^∗∗∗^**
Chlorophyll a	–0.41^∗∗∗^	–0.10	**–0.46^∗^**	–0.29	–0.57^∗∗∗^	–0.23	–0.14	0.04	**–0.26^∗^**
Latitude	**–0.31^∗∗∗^**	**–0.31^∗^**	**–0.59^∗∗∗^**	0.39	–0.33^∗∗^	**–0.55^∗∗∗^**	**–0.40^∗^**	0.34	**–0.29^∗^**
Bacterial abundance	–0.24^∗∗^	–0.08	0.06	0.19	–0.42^∗∗∗^	–0.01	–0.09	0.29	–0.13


*Nitrite, ammonium, and longitude did not exhibit significant relationships with diversity and were not included. ^∗^p-values < 0.05, ^∗∗^p-values < 0.01, ^∗∗∗^p values < 0.001.*

We explored the positive correlation with temperature and salinity further. A plot of Chao1 vs. temperature revealed a negative quadratic relation with the highest species richness for both spring and fall samples converging at a temperature of ∼10°C for all the samples, although this temperature was slightly higher (13°C) for HL (Figure 7A). A closer examination of the salinity and temperature values at 50 m depth along the HL transect revealed that the intermediate temperatures, the shift in salinity and the highest bacterial species richness (Chao1) all coincided with the location of the shelf break, 200 km offshore (Figure 7C–H), a pattern that was accentuated in the fall (Figure 7D,F,H), relative to spring (Figure 7C,E,G) in both 2014 and 2016.

### Beta Diversity of the Bacterial Communities on the Scotian Shelf

#### Correlation With Environmental Factors and Geographic Distance

Bray–Curtis beta diversity was used as a metric to determine the effects of physiochemical gradients and spatial separation on shaping overall community structure similarity on the SS. The separation of community structure in broad categories of season and size fraction, as well as depth in the water column was supported statistically through ANOSIM tests (Supplementary Table S9).

**FIGURE 8:**
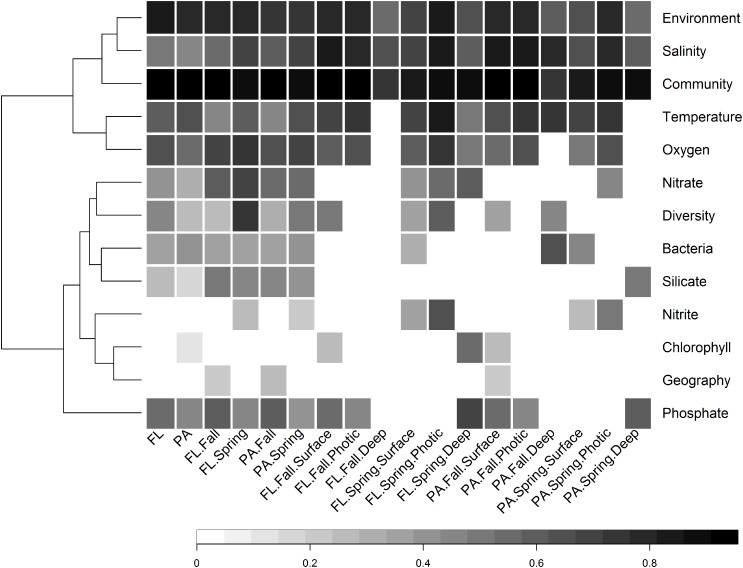
Heatmap of significant Partial Mantel correlation (Spearman’s) between pairwise Bray–Curtis similarity between samples and either pairwise geographic distance between sites, or pairwise environmental dissimilarity between samples, conducted on different subsets of samples. Every Partial Mantel test controlled for the effect of geographic distance between sites. The tests concerning the significance of geographic distance controlled for the environmental distance between sites as determined by the following non-collinear variables: temperature, salinity, oxygen, nitrate, ammonium, and nitrite. Only results that were highly significant (*p* < 0.001) are shown, while non-significant correlations are left blank. The community variable refers to the correlation between the Bray–Curtis community similarity of FL and PA size fractions from the same subset of samples, e.g., fall, or fall photic.

Environmental factors are often spatially related with geographic distance, resulting in sites in close geographic vicinity having similar environmental conditions. We therefore attempted to disentangle the effects of environmental distance from geographic distance on the similarity of bacterial communities using Partial Mantel tests that controlled for the effects of geographic distance while testing for the effects of environmental distance on community structure, and vice versa. Partial Mantel tests were calculated between community dissimilarity matrices and either the entire environmental distance matrix, the geographic distance matrix, or distance matrices generated from individual environmental variables (Figure 8). Each of these tests was performed on either the entire set of FL and PA samples, or on subsets of these samples depending on sampling season and depth, to determine how drivers of community structure changed with different external conditions. The results indicated that overall, environmental variation between sites has a larger influence on community structure than geographic distance (FL: env *r* = 0.84, *p* < 0.001; geo *r* = 0.05, *p* > 0.01; PA: env *r* = 0.79, *p* < 0.001; geo *r* = 0.04, *p* > 0.01; Partial Mantel Tests). The geographic distance between sites was rarely significantly correlated with the bacterial community structure of any subset. Mantel correlation further indicated that while the bacterial community structure of the FL and PA size fractions were significantly different (ANOSIM test: *R* = 0.37, *p* < 0.001; Supplementary Table S9), there was a highly significant positive relationship between the two size fractions (*r* = 0.92, *p* < 0.001; Partial mantel test) (Figure 8).

There was a wide degree of variation in correlations between specific environmental variables and the community structures of different subsets. Salinity was significantly correlated with all subsets; however, the degree of correlation varied from 0.47 in the PA subset, to 0.86 in the FL spring photic subset (Figure 8). Temperature and oxygen were also highly correlated with many subsets. Temperature had the strongest correlation with the entire PA community, while oxygen had the strongest correlation with the entire FL community.

#### Influence of the Bathymetry and Regional Circulation

**FIGURE 9:**
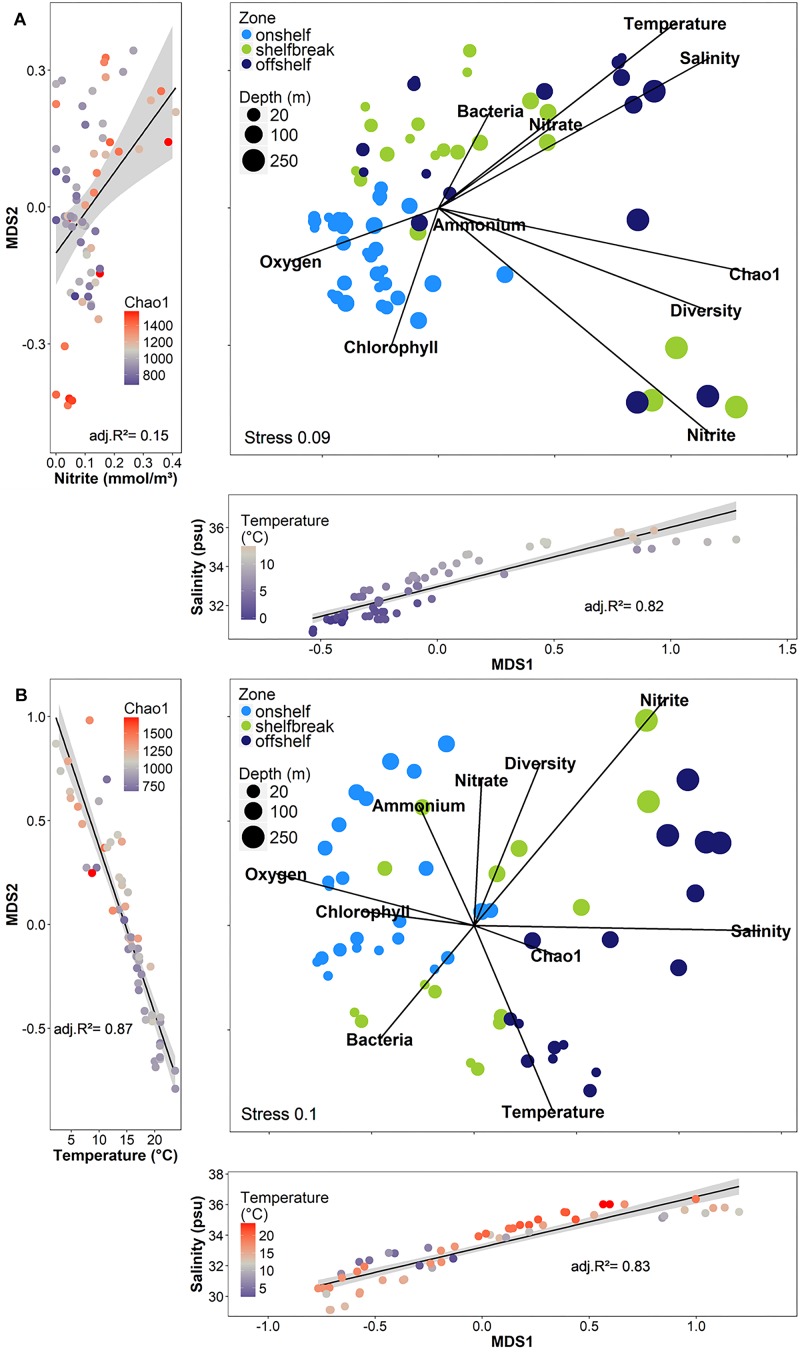
Non-metric multidimensional scaling analysis (NMDS) of Hellinger-transformed free-living OTU abundance data using the Bray–Curtis dissimilarity from 2014 across shelf transects in **(A)** spring **(B)** fall. The strong influences of temperature, salinity, and nitrite (spring only) on the axes of the NMDS are shown in the side and bottom plots. The sizes of the dots for the depth represent a gradual scale from 0 to 250 m.

Although geographic distance did not have a large impact on similarity between bacterial communities, we used the bathymetry of the SS, seawater temperature and salinity, and known circulation patterns in our study region to group the samples into either on-shelf, and off-shelf, with 5 stations assigned to the shelf break group (BBL5, LHB6, HL5.5, HL6, and LL7) based on their physical location and their temperature and salinity features (Figure 1 and Supplementary Figure S1). Analysis of Bray–Curtis dissimilarity of Hellinger transformed bacterial community structure by NMDS and PERMANOVA in spring and fall showed that the on-shelf and off-shelf communities were significantly different from each other in both seasons (Figure 9 and Table 2). In the spring (Figure 9A), the grouping of samples (based on Bray–Curtis dissimilarity) displayed on NMDS plots indicated that highly similar microbial communities were recovered in samples from on-shelf stations, while shelf break and off-shelf stations were not statistically significantly different from each other (Table 2). There was, however, a strong influence of sampling depth, where samples from deep, warm, salty water clustered together (Figure 9A). These small deep-water clusters were also highly diverse based on their Chao1 values (Figure 9A). The fall microbial communities, although less tightly clustered within their assigned geographic categories, were still significantly segregated in their respective groupings, with shelf break microbial communities representing an intermediate between on-shelf and off-shelf communities (Table 2). In fall, microbial community structure similarity was also depth-dependent due to stronger water column stratification in the fall than in the spring, leading to significant clustering of most surface samples, superimposed on the increasing dissimilarity between onshore, shelf break and offshore stations.

**Table 2:** PERMANOVA results of Hellinger-transformed OTU abundance data from select transects by geographic zone using the Bray–Curtis dissimilarity matrix with 1000 permutations.

	DF	*F*	*R*^2^	*p*-adjusted	
**Spring (*F*_2,127_ = 8.89, *p* < 0.001)**				
On-shelf vs. shelf break	97	9.83	0.09	0.003	^∗^
On-shelf vs. off-shelf	89	14.01	0.14	0.003	^∗^
Shelf break vs. off-shelf	71	2.06	0.03	0.132	
**Fall (F_2,108_ = 11.92, *p* < 0.001)**					
On-shelf vs. shelf break	78	7.97	0.09	0.003	^∗^
On-shelf vs. off-shelf	79	20.91	0.21	0.003	^∗^
Shelf break vs. off-shelf	62	5.91	0.09	0.003	^∗^


*Pairwise comparisons used Bonferroni correction. ^∗^ indicates significance at α = 0.05. Spring and fall were significantly different (F_*1,239*_ = 42.27, p < 0.001)*.

**FIGURE 10:**
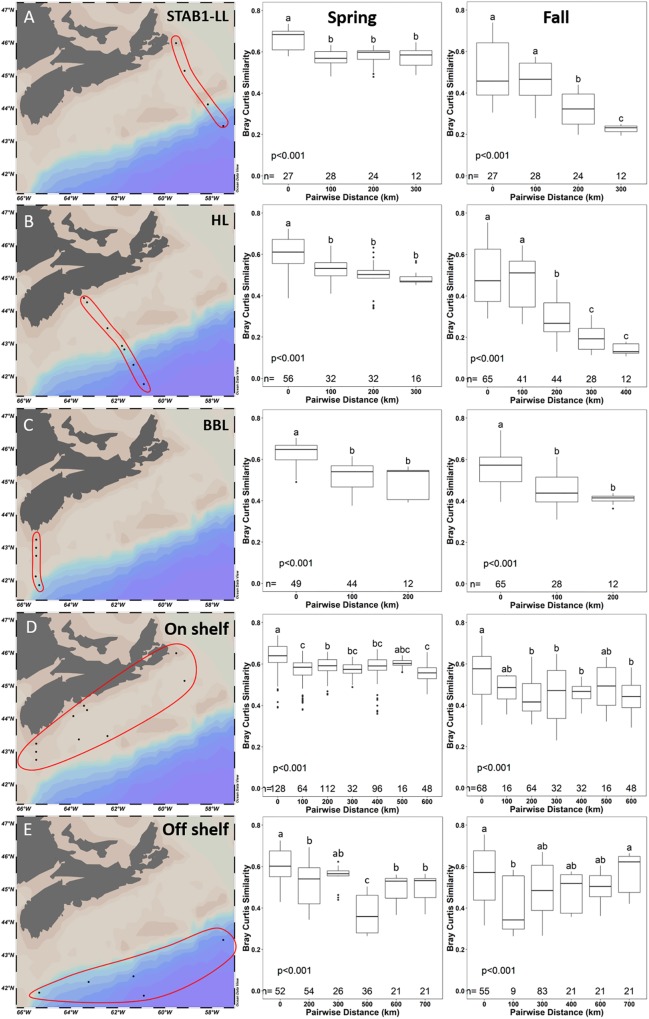
Pairwise comparisons of Bray–Curtis similarity between samples in 100 km bins along 2014 cross-shelf transects (**A–C**: STAB1-LL, HL, and BBL, respectively) and from near- and offshore samples (**D,E**, respectively). Significance between distance bins was tested by ANOVA with *post hoc* testing by Tukey tests (α = 0.05). Bins not sharing a letter are significantly different. The numbers (*n*=) at the bottom of the graphs represent the number of samples that were compared at each distance stated on the *x*-axis. Samples from 1 to 100 m depth were used.

To further investigate the effect of on-shelf and off-shelf similarity patterns observed in Figure 9, we calculated Bray–Curtis dissimilarity-distance decay curves of the microbial communities across three longitudinal transects from on-shelf to off-shelf (LL in the north, HL in the middle shelf, and BBL in the South) (Figure 10A–C) with samples from 1 to 100 m. Similarly, Bray–Curtis dissimilarity-distance decay curves were obtained for stations grouped into on-shelf and off-shelf regions (Figure 10D,E) as previously defined in Supplementary Figure S1. On-shelf stations are within the path of the Nova Scotia Current (NSC) and off-shelf stations are within the path of the Shelf Break Current (SBC). The NSC and SBC contribute to the general circulation pattern of the SS and Scotian Slope, respectively, with the water flowing from the NE to SW along the SS. As expected, statistically significant differences in mean salinity, temperature, as well as in oxygen, phosphate and chlorophyll concentrations were observed between on shelf and off shelf samples (Supplementary Figure S7 and Supplementary Table S7). However, with the exception of fall off shelf, environmental parameters were also variable within the on shelf and off shelf groups of samples (Supplementary Figure S8). Based on Bray–Curtis dissimilarity values, there were significant changes in the spring bacterial community samples compared pairwise along all three across-shelf transects, with the similarity of microbial communities significantly decreasing with increasing distance from each other (Figure 10). The pattern of decreasing similarity with distance between samples was much more pronounced in the fall across shelf transects, where we observed an abrupt significant decrease in similarity at a 200 km pairwise distance that likely reflected the presence of distinct microbial communities on opposite sides of the shelf break (which is on average located at 200 km distance from the shore) (Figure 10A–C). This pattern of decreasing community similarity was most pronounced for the HL fall transect (Figure 10B), which reached the furthest beyond the shelf break. In contrast, on-shelf and off-shelf communities showed high similarity within seasons, with no observable decreasing trend, through a much broader geographic distance covering up to 700 km (Figure 10D,E). Notably, the bacterial communities of the nearshore samples in the spring had a high Bray–Curtis similarity index along a 600 km distance, although statistically significant differences were still detected (Figure 10D).

Examination of the 10 OTUs with the highest relative abundance in each of the select transects for on- and off-shelf regions showed that the dominant taxa varied between seasons (Supplementary Table S8) and were often among the indicator OTUs listed in Supplementary Tables S5, S6. A few of the dominant OTUs were recovered only from one season in either region. In spring, the taxa *Polaribacter* sp. and Colwelliaceae were dominant in the bacterial community from on-shelf, while SAR86 was found off-shelf. In contrast, *Synechococcus* and *Prochlorococcus* were specifically found in the fall. As seen in the surface temperature and salinity distributions in the fall climatology (Figure 1 and Supplementary Figure S2), OTUs assigned to *Synechococcus* populated the warm shelf waters, but were displaced by *Prochlorococcus* at the sharp salinity boundary at around 43°N (Figure 6). Other notable Alphaproteobacteria that dominated off-shelf in the fall belonged to Erythrobacteraceae and Methylobacteriaceae. Some taxa, however, maintained high relative abundance in one season throughout the whole region, while receding in relative abundance in either spring or fall. Four OTUs assigned to Rhodobacteriaceae spp. and Oceanospirillales sp. showed high relative abundance throughout the bacterial communities in the spring, but were found in high relative abundance only on the on-shelf communities in the fall. A reverse pattern was observed for OTUs assigned to Pelagibacteriaceae and *Alteromonas* sp., where their high relative abundance off-shelf in the spring was extended to the whole region in the fall.

## Discussion

Several global studies have concluded that, with the exception of a few cosmopolitan species, marine microbes display broad biogeographic distribution patterns in the ocean (Ghiglione et al., 2012; Milici et al., 2016b). In a recent global survey of the ocean microbiome, temperature was identified as the most significant environmental factor driving microbial community composition (Sunagawa et al., 2015), raising the question of what processes are involved in maintaining the observed diversity of microbial communities. Microbial processes, and thus microbial community composition, are responsive to their environment on timescales of days to weeks (e.g., phytoplankton blooms) and at the seasonal and interannual timescales. Spatially, microbial community composition varies at the microscale as the result of aggregation, vertically on scales of meters, or at the ocean basin scale over 1000 s of km.

Although surface waters of ocean gyres are relatively homogenous in their physicochemical properties over thousands of km, regions with steep horizontal gradients can form at spatial scales of 100–200 km or less when two distinct water masses meet (e.g., fronts). As shown here and in previous studies (Baltar et al., 2016; Djurhuus et al., 2017), these boundaries are important regionally in determining microbial community composition. In our study, the bacterial community of the Scotian Shelf (SS) was analyzed by high throughput amplicon sequencing of the 16SrRNA gene V6–V8 variable region from a total of 451 DNA samples collected during spring and fall cruises in two separate years. Based on the relevant scales of variability presented above, we discuss below the dominant members of the bacterial communities, the potential roles of indicator species, the seasonal differences in on-shelf and off-shelf bacterial communities and finally the processes at play in shaping community composition.

### Bacterial Community Composition in FL and PA Fractions

In general agreement with findings of previous studies (Crespo et al., 2013; Ganesh et al., 2014; Mohit et al., 2014; Rieck et al., 2015; Milici et al., 2016a), the taxa with high relative abundance in the PA fraction belonged to Deltaproteobacteria, Flavobacteriia, Verrucomicrobiae, Saprospirae (now classified with Bacteroidetes), and OM190 (from the Planctomycetes phylum), while Alphaproteobacteria and SAR406 clades were enriched in the FL fraction. Pelagibacteraceae, Rhodobacteraceae, and Bacteroidetes, the main taxa recovered in the FL fraction, are known as dominant marine bacteria in temperate coastal waters (Gilbert et al., 2012; El-Swais et al., 2015; Xiaomin et al., 2015). As expected, OTUs belonging to Pelagibacteraceae (also known as SAR11), some of the smallest but most abundant organisms on the planet (Morris et al., 2002; Giovannoni, 2017; Zhao et al., 2017), were dominant members of the bacterial communities in our study, however, with strong evidence for niche partitioning (Brown et al., 2012; Grote et al., 2012; Eren et al., 2013). Pelagibacteraceae OTUs were key indicators for FL, surface, photic, deep, off-shore, on shore, in both spring and fall. Specifically, fall and spring communities were dominated by Pelagibacteraceae ecotypes of the tropical PIa.3 (OTU#307744) and polar P1a.1 (OTU#637092) clades, respectively (Morris et al., 2005; Brown et al., 2012; Salter et al., 2015).

In contrast to seawater, particles provide a heterogeneous habitat for their associated bacteria (Alldredge and Cohen, 1987; Alldredge and Silver, 1988; Simon et al., 2002; Wright et al., 2012). Most PA bacteria are assumed to be attached either to marine snow, or other plankton, especially during blooms. Steep gradients in nutrients and oxygen create regions of microscale oxyclines in particles, resulting in selection for anaerobic bacteria and the concomitant buildup of anoxic metabolites such as hydrogen sulfide and methane, in an otherwise oxygenated environment (Shanks and Reeder, 1993). Populations of bacteria with upward of a thousand times more cells than in a comparable volume of seawater have been observed on particles (Alldredge et al., 1986; Turley and Mackie, 1994; Simon et al., 2002). Furthermore, aggregated particulate matter is more likely to contribute to transport of material from the surface to deep ocean (Boyd and Newton, 1999; Mestre et al., 2018) accounting for a larger proportion of carbon to the biological pump than FL bacteria. The relatively abundant uncultured class of Planctomycetes OM190 recovered in the PA fraction throughout the samples has been found in association with macroalgae (Lage and Bondoso, 2014). A member of the OM60 clade (OTU#630330), with high relative abundance in the PA fraction, is a likely representative of bacteriochlorophyll-containing aerobic anoxygenic phototrophs (AAPs). Most of the AAP bacteria isolated in culture belong to the OM60 clade (Zheng et al., 2016) and *Congregibacter litoralis*, a cultured member of this clade, is known for its ability to aggregate (Spring et al., 2009). The class of Cyanobacteria (containing *Synechococcus*) was also noticeably more abundant in the PA-associated fraction. The presence of *Synechococcus*, a small, oligotrophic, free-living genus, in the PA size fraction has been observed before, suggesting that it can also be bound to particles or hosts (Turley and Mackie, 1994; Simon et al., 2002; Crespo et al., 2013; Jackson et al., 2014; Yung et al., 2016). The differences in community composition between FL and PA fractions we observed is in agreement with previous studies, and supports the distinct functional roles of these communities (Cram et al., 2015; Milici et al., 2017). We thus advocate for size fractionation when sampling aquatic habitats.

### Bacterial Communities in Surface, Photic and Deep Waters

Several bacterial OTUs were preferentially recovered from specific zones within the water column vertical profile. On the SS, surface waters were inhabited by Cyanobacteria, AAP bacteria, rhodopsin-containing bacteria, and bacteria associated with phytoplankton (e.g., Flavobacteriia). Below the photic zone, deep samples contained Deltaproteobacteria, Acidobacteria, and candidate phyla (e.g., PAUC34f), as well as select OTUs of Pelagibacteraceae. Deltaproteobacteria are diverse metabolically, with the ability to conduct sulfur oxidation, carbon fixation, C1 utilization, and heterotrophy (Sheik et al., 2014), pathways that often dominate in deeper water. Marine Group A (SAR406) and Planctomycetes, with members that participate in anammox and sulfur cycling (Francis et al., 2007; Wright et al., 2014), were significantly more abundant in deep waters. Further metagenomics and metatranscriptomic studies of the deep waters of the SS would provide information on whether these taxa are active members of the community or if they are dormant and resuspended by chance (Nemergut et al., 2013), especially in the shallow shelf region.

### Spring and Fall Bacterial Communities in On-Shelf and Off-Shelf Waters

The boundaries between the on- and off-shelf microbial communities are delimited by the general circulation of the SS, which results in highest dispersal rates along the coast of Nova Scotia due to rapid flow of the Nova Scotia Current (NSC) and lower dispersal rates at and beyond the shelf-break current (SBC) (Rutherford and Fennel, 2018). The bacterial taxa preferentially found in either the spring or the fall most likely responded to differences in nutrients and productivity between seasons. On the SS, eukaryotic phytoplankton blooms and highly productive conditions are associated with the spring, whereas the early fall is associated with warm, stratified, and nutrient-limited conditions (Craig et al., 2014; Dasilva et al., 2014; Li et al., 2006). Bacteria preferentially found in the spring are likely copiotrophs able to achieve high growth rates in favorable conditions, whereas bacteria preferentially found in the fall could be considered oligotrophs with growth strategies adapted to low nutrients (Lauro et al., 2009). Our results show that the microbial community compositions of on-shelf and off-shelf regions are defined both by season and by their geographical location relative to the shelf break. However, we observed persistence at high relative abundance of select spring bacterial taxa during the fall, albeit only on-shelf. Conversely, a few bacterial taxa with high relative abundance throughout the SS during the fall were also significant members of the spring community, but restricted to the off-shelf region (Supplementary Table S8). These observations suggest that there may be an endemic population for select taxa throughout the SS waters, ready to grow under appropriate environmental conditions, supporting the view that the environment selects (Baas Becking, 1934). Alternatively, water exchange between the water masses, albeit low (Rutherford and Fennel, 2018), may nevertheless be sufficient to recruit bacterial taxa across the shelf break.

In the spring, the most abundant OTU was the cold-water ecotype of *Pelagibacter* clade P1a.1 (OTU#637092). *Polaribacter* and Colwelliaceae were also found in high relative abundance on-shelf only. Taxa more abundant in spring samples are likely associated with either phytoplankton directly via symbiosis or other cell-to-cell interactions, or indirectly, exploiting the dissolved organic carbon leaking from the phytoplankton blooms (Teeling et al., 2012; Georges et al., 2014; Wear et al., 2015). Many OTUs from Flavobacteriia, including those from *Ulvibacter* and *Polaribacter*, were strongly associated with the spring season and have previously been identified as important genera in the succession of phytoplankton blooms (Teeling et al., 2012; Klindworth et al., 2014; El-Swais et al., 2015). Cyanobacteria were the main taxa found more often in the fall. Minimalist oligotrophic cyanobacterial groups like *Prochlorococcus* and *Synechococcus* are known to thrive in extremely low-nutrient environments (Partensky and Garczarek, 2010; Flombaum et al., 2013). Therefore, the presence of these genera in the SS region highlights the extent of temperature stratification and oligotrophic conditions in the early fall. Notably, *Prochlorococcus* reached high relative abundances only in the off-shelf regions at any time during the sampling. Photosynthetic bacteria belonging to the AAP, as well as methylotrophs, were in high relative abundance in the off-shelf region of the SS, indicating that this region supported a bacterial community with a more specialized metabolism to exploit limited environmental resources.

### Patterns of Bacterial Alpha Diversity in FL and PA Fractions

Alpha diversity, measured as both species evenness and species richness, was significantly higher in the PA fraction than the FL fraction (Supplementary Figure S6). With some exceptions (e.g., Hollibaugh et al., 2000), a number of studies have reported similar trends (Crespo et al., 2013; Ortega-Retuerta et al., 2013; Rieck et al., 2015; Yung et al., 2016). Higher diversity in the PA fraction could be explained by the microenvironments of particles allowing for the accumulation of functionally diverse species, responding to microscale environmental gradients. The observed correlation between FL and PA diversity (Supplementary Figure S6), especially with species richness, may be a consequence of the broad range of environments sampled in this study. Although we cannot currently identify the processes that led to this correlation, our observations suggest that environmental factors affecting diversity are similar for FL and PA fractions.

Alpha diversity was strongly correlated with several environmental factors (Table 1). Teasing apart the role of individual environmental factors affecting the diversity and composition of bacterial communities is challenging because of the degree of correlation between temperature, salinity and other factors such as light, nutrients and dissolved gasses. Although correlation does not imply causality, a few statistically significant correlations are worth mentioning. In particular the negative relationship observed between chlorophyll *a* and diversity in our study is in agreement with previous observations that spring blooms and highly productive areas have lower bacterial diversity (i.e., are dominated by a few opportunistic species) (Wemheuer et al., 2014). The negative correlation between bacterial diversity and dissolved oxygen may also be linked to the lower diversity associated with spring bloom conditions, where high primary production leads to high dissolved oxygen concentration in the photic zone. While dissolved oxygen ranged between 130 and 350 μmol/kg, concentrations well above the suboxic levels indicative of strong oxygen minimum zones (OMZ), lower oxygen concentrations below the photic zone partially reflect the respiration of labile organic matter, likely favoring microbial communities with diverse alternative metabolic approaches to acquire energy and nutrients, and could explain the trends seen here (Wright et al., 2012). Several other studies have observed similar inverse relationships between oxygen concentration and bacterial diversity, with the most diverse bacterial communities found at low, but still measurable, oxygen concentrations (Zaikova et al., 2010; Beman and Carolan, 2013; Spietz et al., 2015; Walsh et al., 2015; Wang et al., 2015).

In our study, bacterial diversity on the SS was positively correlated with salinity over a range of 28–36 PSU. Others found a negative relationship between salinity (in the range of 12–33 PSU) and bacterial diversity in a regional surface study from the East China Sea (Wang et al., 2015), while studies targeting estuarine systems (salinity ranges between 0 and 35 PSU) observed a bimodal relationship with high diversity in both the freshest and the saltiest environments in the system, with minimum diversity in estuarine waters (Fortunato et al., 2012; Campbell and Kirchman, 2013). In contrast, a global compilation of oceanic sites (Milici et al., 2016b) found no effect between salinity ranging between 33 and 37.5 PSU and bacterial diversity. There is currently no agreement on the relationship between salinity and bacterial alpha diversity, and most likely the observed variability of this relationship reflects specific regional features rather than global trends in microbial diversity patterns, as is discussed below for our study area (Figure 7).

Although the correlation between alpha diversity and temperature was not as strong as for other environmental parameters in our study, we further examined this relationship because it has been extensively implicated as a determinant of marine microbial diversity patterns in several global surveys (Ghiglione et al., 2012; Sunagawa et al., 2015; Milici et al., 2016b), and temperature is a critically important environmental factor in the context of climate change (Wethey and Lima, 2012). In our study, the relationship between temperature and species richness was complex (Figure 7A), showing a positive correlation between temperature and Chao1 in spring and a negative one in the fall. However, combining spring and fall samples yielded a relationship fitted by a second order polynomial model with maximum species richness at intermediate temperatures of 13°C for the HL transect (Figure 7A). We showed that the intermediate temperature and the highest species richness are both coincident with the shelf break (Figure 7) at the confluence of the two main southward-flowing currents of the SS (Drinkwater and Gilbert, 2004). While several processes could lead to the observation of maximum species richness at intermediate water temperature, our results (Figure 7) support the view that the highest species richness of microbial communities on the SS is associated with the boundary between two water masses at the shelf break. Based on the overlap between on-shelf, off-shelf, and shelf break OTUs, we determined that less than 4% of the OTUs recovered from the shelf break stations were unique, while this proportion was 25% in both the on-shelf and off-shelf regions. This suggested that the increased diversity observed at the confluence of the water masses is largely an additive effect caused by mixing of the distinct on-shelf and off-shelf communities (Supplementary Figure S9). Similar processes may have led to the temperature-diversity relationship displaying highest alpha diversity of microbial communities at intermediate temperatures (∼15°C) observed in some recent global surveys of microbial diversity (Sunagawa et al., 2015; Milici et al., 2016b). However, sampling resolution at the ocean basin or global scale, may currently not be high enough spatially and temporally to provide estimates of alpha diversity of microbial communities at the transition zones where water masses of different temperature and salinity meet.

### Patterns of Beta Diversity in Microbial Communities of the Scotian Shelf Region

Our results support the cosmopolitan “environment selects” theory and are in line with other regional marine microbial studies from different locations (Jiang X. et al., 2012; Nguyen and Landfald, 2015; Wang et al., 2015). There was a strong correlation between the community structure of PA and FL size fractions, suggesting that biological interactions may be important in shaping bacterial communities in this region. Strong biological interactions have previously been identified as drivers of global marine community structure (Lima-mendez et al., 2015), and these relationships could be attributed to a number of underlying processes such as cross-feeding, environmental preferences, predation, or symbiosis (Fuhrman et al., 2015; Needham and Fuhrman, 2016).

Of the individual environmental variables tested, temperature and oxygen were the environmental factors most highly correlated with community similarity. Regionally, temperature has been identified as a strong driver of bacterial community structure temporally (El-Swais et al., 2015), and of eukaryotic phytoplankton community structure on the SS at a regional spatial scale (Li and Harrison, 2008; Dasilva et al., 2014), as well as globally (Rusch et al., 2007; Ladau et al., 2013; Sunagawa et al., 2015). Oxygen, previously identified as a key driver of community composition (Stewart et al., 2012; Wang et al., 2015), is extremely important in defining bacterial niches (Wright et al., 2012), and the presence or absence of oxygen in the marine environment can dramatically change the type of microorganisms and the types of metabolic processes occurring (Ganesh et al., 2014; Hawley et al., 2014). The expected trends of decreasing oxygen and increasing temperature associated with climate change (Finkel et al., 2010; Wright et al., 2012; Schmidtko et al., 2017) will have strong implications for the future of bacterial communities on the SS.

Our intensive sampling of the SS allowed us to identify the importance of the shelf break and of regional-scale circulation patterns (Rutherford and Fennel, 2018) on the bacterial community composition observed in spring and fall. The patterns of diversity observed in the bacterial community supported the view that the shelf circulation and its associated frontal zone create a physical boundary that reduces dispersal between off-shelf and on-shelf regions (Figure 7, 9, 10), leading to highest species richness near the shelf break (Figure 7) and distinct bacterial communities on either side (Figure 9 and Table 2). The Bray–Curtis decay curves of three across-shore transects show that similarity between samples decreases significantly at a paired distance of 200 km, especially in the fall (Figure 10). This decrease in the similarity of bacterial communities is most pronounced for the HL transect that covers a 400 km distance across the shelf and with stations distributed evenly across the shelf break (Figure 10B). In contrast, the high similarity of the spring bacterial communities over a distance of >600 km in the near-shore transect is notable. To explain these observations, we propose that the high flow rates of the NSC in the spring resulted in high dispersal rates along on-shelf transect that homogenized the bacterial community. The results also indicate an important role for dispersal and dispersal limitation in our study, as demonstrated by the distance-decay curves of across-shelf sections compared to those of on-shelf and off-shelf regions (Figure 10) that follow the boundary between the major currents on the SS, restricting water exchange across the shelf break. Although our results do not rule out a significant role for environmental selection, the marked decrease in community similarity between on-shelf and off-shelf stations, reflected by the marked decrease in Bray–Curtis similarity at a paired distance of 200 km distance, suggests that a physical, although fluid, barrier develops at the confluence of the prevailing currents (Rutherford and Fennel, 2018), supporting the role of dispersal limitation in the development of distinct on-shelf and off-shelf bacterial communities (Figure 10). In contrast, the results showing no decay of similarity over a much larger distance on the on-shelf and off-shelf regions imply a role for dispersal in homogenizing the bacterial community faster than the environment can modify them. The effects of dispersal were most noticeable in the spring when the NSC is stronger than in the fall (Rutherford and Fennel, 2018). Although the fluidity of oceanic environment limits the degree of dispersal limitation, thereby reducing also the effect of historical processes in shaping overall bacterial community structure (Martiny et al., 2006), dispersal in the ocean is controlled to a large extent by major ocean currents and will therefore be directional, as seen in our results (Figure 10), rather than simply related to the geographic distance (Lindström and Langenheder, 2012).

Our study, conducted at the regional scale with high resolution sampling across strong environmental gradients, covers a region of the temperate Western North Atlantic at the junction of the Arctic Gateway to the northeast and the Gulf of Maine to the southwest, with warm waters of the Gulf Stream bordering to the east. The results of our 2-year study are compatible with reports that the long-term composition of marine microbial communities is stable despite short-term variation (Cram et al., 2015; Fuhrman et al., 2015). Together, these findings indicate that similar microbial assemblages are expected on the SS on an annual basis. However, the sharp delineation of the *Prochlorococcus* northern distribution range and the continued reports of water temperature increase in the region, as well as earlier onset of spring warming (Wethey and Lima, 2012), point to the importance of time-series observations into the future to allow the detection of early change in the presently predictable microbial community structure in response to environmental climate change shifts such as increased temperatures, ocean acidification, or decreased salinity due to Arctic melt (Hutchins and Fu, 2017).

## Conclusion

This study provides a characterization of the bacterial community of the SS in the Northwest Atlantic Ocean. In addition to the detailed regional observations, our study also integrated spatial oceanographic features of the SS, depth, and seasons, to determine the factors most correlated with changes in microbial community structure and biodiversity across multiple dimensions. Multivariate analysis identified temperature, salinity and dissolved oxygen as important environmental factors correlated with microbial community structure. Many bacterial OTUs were preferentially found in specific seasons, depths, or size fractions, and contributed to distinct bacterial assemblages, which reoccurred over two separate years. We showed that the bathymetry of the SS and the dominant ocean currents lead to the development of on-shelf and off-shelf bacterial communities, with the highest species richness observed at the shelf break, where two water masses come in contact. The higher species richness observed at the shelf break is most likely the result of mixing between the on-shelf and off-shelf microbial communities. Environmental gradients, and thus contemporary selection, had a stronger effect on shaping community structure than historical processes such as dispersal limitation within the on- and off-shelf bacterial communities. However, the circulation patterns and major ocean currents of the regions restricted dispersal of on-shelf and off-shelf bacterial communities across current boundaries. Our study provides a detailed baseline description of the bacterial community structure on the Scotian Shelf. Repeated observations in this region, combined with the present observations, will be instrumental in assessing the effect of environmental changes such as warming temperatures and reduced oxygen on microbial community structure and function.

## Author Contributions

JZ conceived the study, collected samples, extracted DNA, analyzed the sequence data, and wrote the manuscript with input from co-authors. CW collected samples, extracted DNA, analyzed sequence data, and wrote sections of the Results and Discussion. AC and ML performed Illumina 16S rRNA amplicon sequencing, and contributed to the processing of the sequence reads. CJ and WL provided the oceanographic data for the study and commented on version of the manuscript. JLR conceived the study, contributed to the data analysis, wrote the manuscript with JZ and CW, and revised the manuscript with JZ and CW.

## Conflict of Interest Statement

The authors declare that the research was conducted in the absence of any commercial or financial relationships that could be construed as a potential conflict of interest.
