# Supplementary material for: Drivers of Regional Bacterial Community Structure and Diversity in the Northwest Atlantic Ocean
Source: Front Microbiol. 2019 Feb 21;10:281. doi: 10.3389/fmicb.2019.00281 (PMC6393369; doi:10.3389/fmicb.2019.00281)
Supplement: Supplementary file 1 [file Data_Sheet_1.PDF]

Supplementary Material

Drivers of regional bacterial community structure and diversity in the Northwest Atlantic Ocean

Jackie Zorz[1,□], Ciara Willis[1], André M. Comeau[2], Morgan G. I. Langille[2], Catherine L Johnson[3], William Li[3], Julie LaRoche[1]*

[1]Department of Biology, Dalhousie University, Halifax, Nova Scotia, B3H 4R2, Canada
[2]CGEB-Integrated Microbiome Resource (IMR and Department of Pharmacology, Dalhousie University, Halifax, Nova Scotia, B3H 4R2, Canada
[3]Fisheries and Oceans Canada, Bedford Institute of Oceanography, Dartmouth, Nova Scotia, Canada
□ Department of Geoscience, University of Calgary, Calgary, AB, Canada

*Corresponding Author: Phone: (902) 494-4249
E-mail: Julie.LaRoche@dal.ca

Running Title: Bacterial community structure of the Scotian Shelf

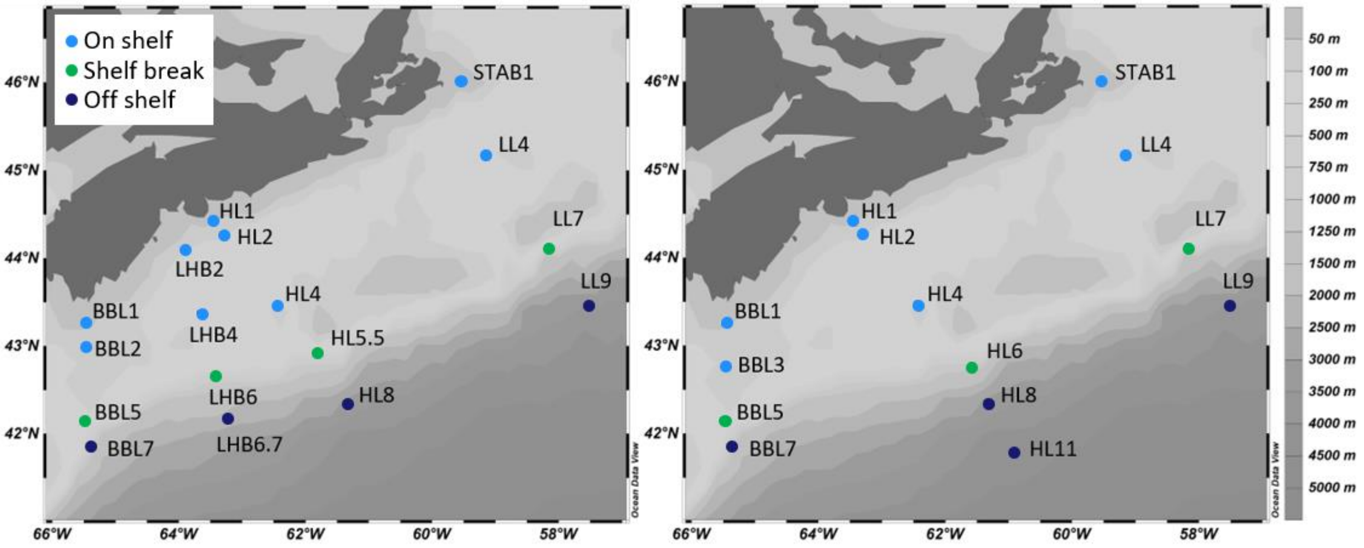

Fig S1. Map of 2014 across shelf transects with zone (on shelf, shelf break, off shelf) indicated by colour.

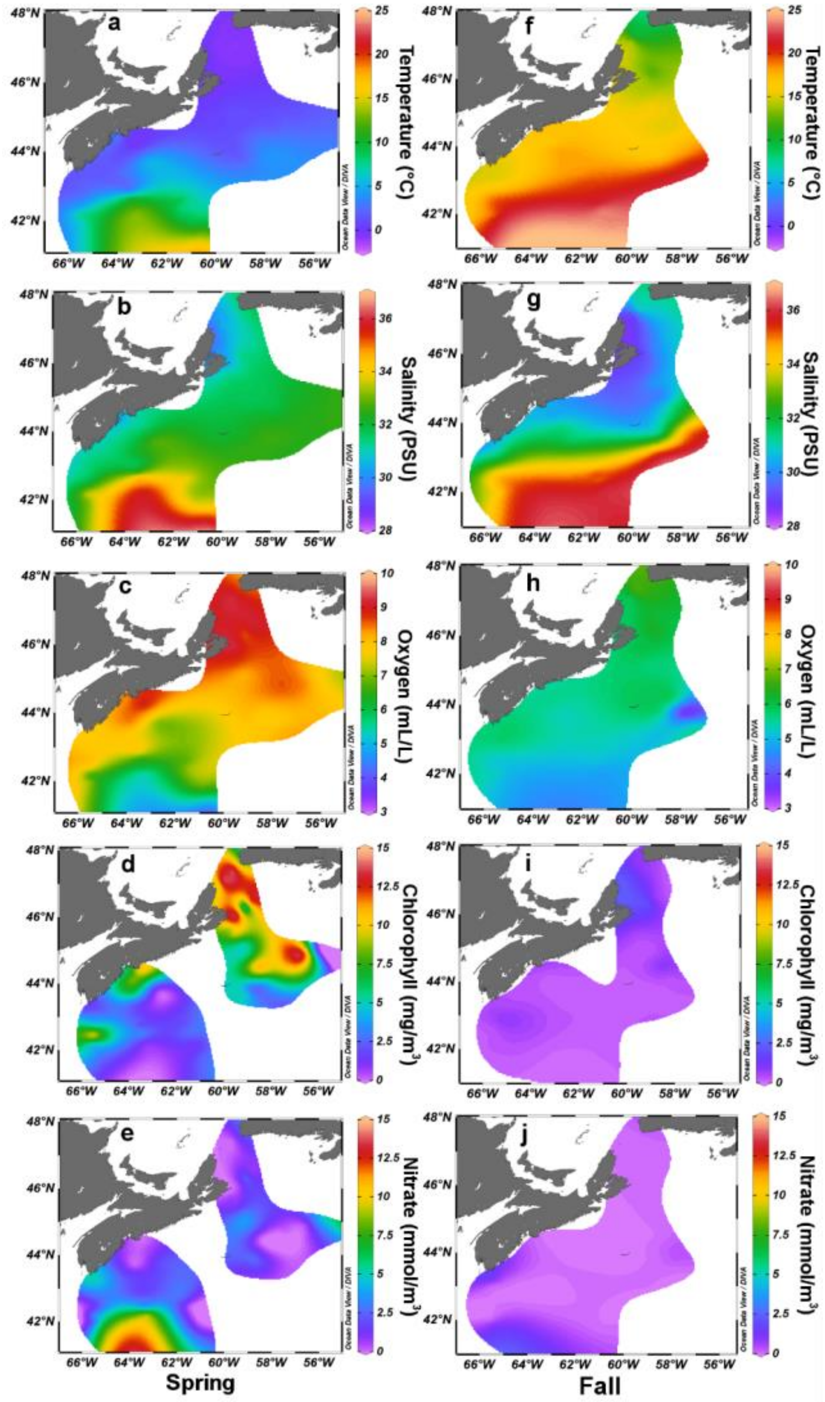

Figure S2 Surface plots of data from spring 2014 (a-e) and fall 2014 (f-j) cruises. Environmental variables displayed include: Temperature (a,f), Salinity (b,g), Oxygen concentration in mL/L (c,h), Chlorophyll concentration in mg/m^3 (d,i), and Nitrate concentrations in mmol/m^3 (e,j). Slightly different cruise tracks resulted in slightly different coverage of the Scotian Shelf between spring and fall cruises.

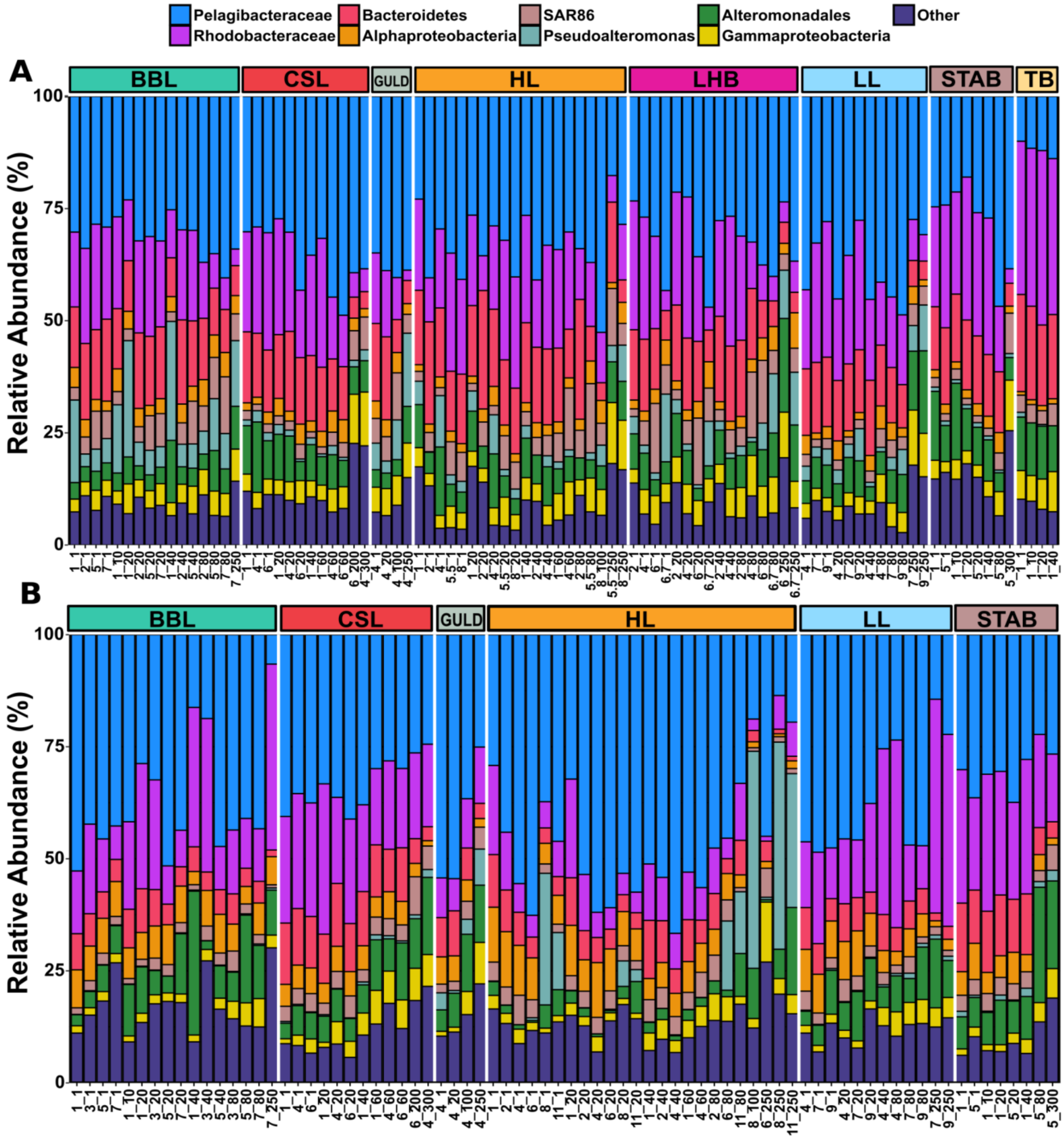

Figure S3. Relative abundance of the most abundant taxa in the free living size fraction on the Scotian Shelf from Spring (A), and Fall (B). Samples are ordered first based on transect, which is displayed along the top of the graph, and then based on depth. The first number along the x-axis refers to the sample station, and the second number refers to the sample depth. Transects: BBL – Browns Bank Line; LHB – LaHave Basin Line; HL – Halifax Line; TB – Thebaud Platform Station; GULD – Gully Station; LL – Louisbourg Line; STAB – St. Anns Bank Line; CSL – Cabot Strait Line.

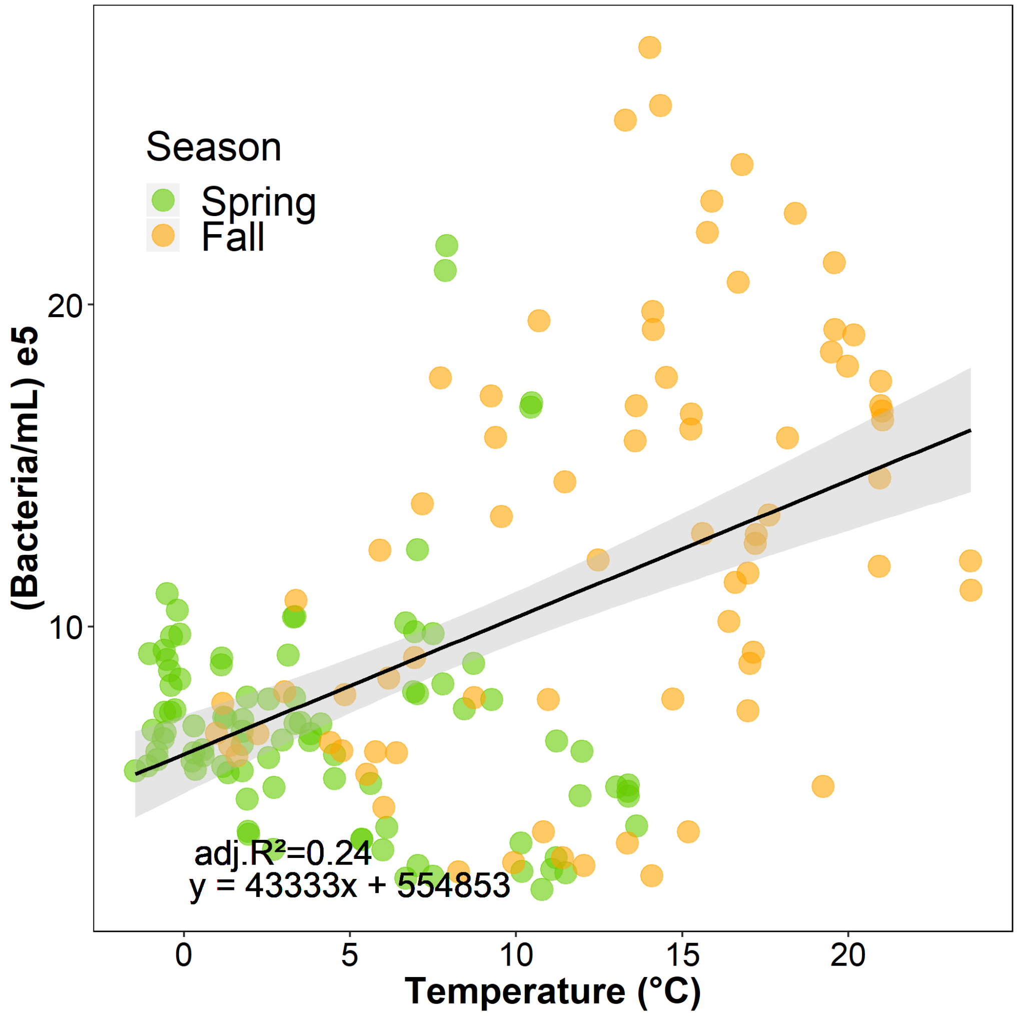

Fig. S4 Correlation of bacterial cell density with temperature across 2014 samples (n=168).

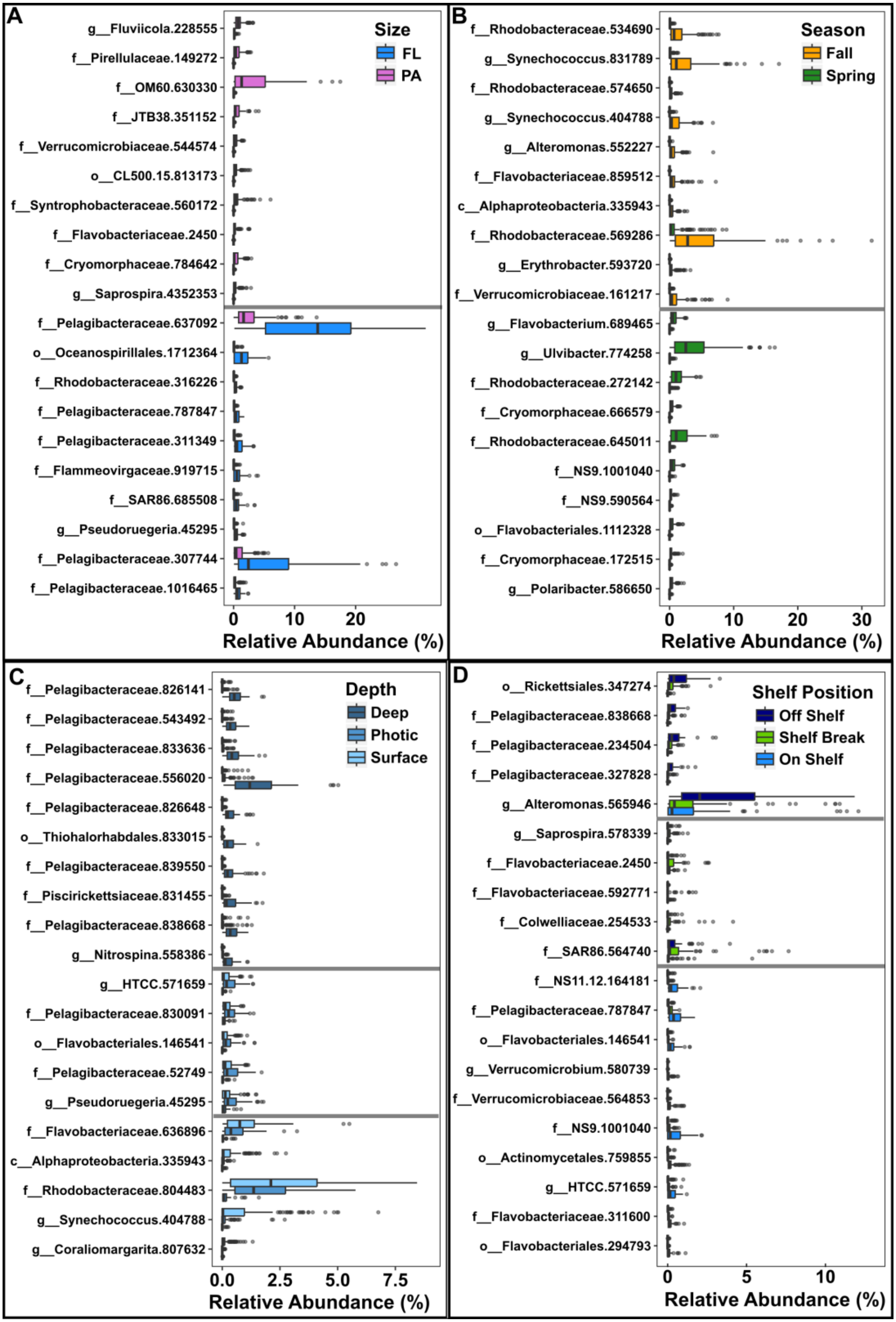
A
Size
FL
PA
g__Fluviicola.228555
f__Pirellulaceae.149272
f__OM60.630330
f__JTB38.351152
f__Verrucomicrobiaceae.544574
o__CL500.15.813173
f__Syntrophobacteraceae.560172
f__Flavobacteriaceae.2450
f__Cryomorphaceae.784642
g__Saprospira.4352353
f__Pelagibacteraceae.637092
o__Oceanospirillales.1712364
f__Rhodobacteraceae.316226
f__Pelagibacteraceae.787847
f__Pelagibacteraceae.311349
f__Flammeovirgaceae.919715
f__SAR86.685508
g__Pseudoruegeria.45295
f__Pelagibacteraceae.307744
f__Pelagibacteraceae.1016465
0
10
20
Relative Abundance (%)
B
Season
Fall
Spring
f__Rhodobacteraceae.534690
g__Synechococcus.831789
f__Rhodobacteraceae.574650
g__Synechococcus.404788
g__Alteromonas.552227
f__Flavobacteriaceae.859512
c__Alphaproteobacteria.335943
f__Rhodobacteraceae.569286
g__Erythrobacter.593720
f__Verrucomicrobiaceae.161217
g__Flavobacterium.689465
g__Ulvibacter.774258
f__Rhodobacteraceae.272142
f__Cryomorphaceae.666579
f__Rhodobacteraceae.645011
f__NS9.1001040
f__NS9.590564
o__Flavobacteriales.1112328
f__Cryomorphaceae.172515
g__Polaribacter.586650
0
10
20
30
Relative Abundance (%)
C
Depth
Deep
Photic
Surface
f__Pelagibacteraceae.826141
f__Pelagibacteraceae.543492
f__Pelagibacteraceae.833636
f__Pelagibacteraceae.556020
f__Pelagibacteraceae.826648
o__Thiohalorhabdales.833015
f__Pelagibacteraceae.839550
f__Piscirickettsiaceae.831455
f__Pelagibacteraceae.838668
g__Nitrospina.558386
g__HTCC.571659
f__Pelagibacteraceae.830091
o__Flavobacteriales.146541
f__Pelagibacteraceae.52749
g__Pseudoruegeria.45295
f__Flavobacteriaceae.636896
c__Alphaproteobacteria.335943
f__Rhodobacteraceae.804483
g__Synechococcus.404788
g__Coraliomargarita.807632
0.0
2.5
5.0
7.5
Relative Abundance (%)
D
Shelf Position
Off Shelf
Shelf Break
On Shelf
o__Rickettsiales.347274
f__Pelagibacteraceae.838668
f__Pelagibacteraceae.234504
f__Pelagibacteraceae.327828
g__Alteromonas.565946
g__Saprospira.578339
f__Flavobacteriaceae.2450
f__Flavobacteriaceae.592771
f__Colwelliaceae.254533
f__SAR86.564740
f__NS11.12.164181
f__Pelagibacteraceae.787847
o__Flavobacteriales.146541
g__Verrucomicrobium.580739
f__Verrucomicrobiaceae.564853
f__NS9.1001040
o__Actinomycetales.759855
g__HTCC.571659
f__Flavobacteriaceae.311600
o__Flavobacteriales.294793
0
5
10
Relative Abundance (%)

Figure S5. List and relative abundance of the 10 most statistically significant indicator species for A) different size fractions, B) seasons, C) sampling depth and D) location on the shelf. A) The top ten most significant indicator species for each size fraction are shown, PA in the upper panel and FL in the bottom panel. B) The top ten most significant indicator species for each season is shown, fall in the top panel, spring in the bottom panel. C) The top ten most significant indicator species for the deep zone, and the five most significant indicator species for the surface and photic zones are shown, deep zone is in the top layer, photic zone is in the middle layer, and surface zone is in the bottom layer. D) The top 10 indicator species for each of off shelf (top), shelf break (middle) and on shelf (bottom) are shown. OTUs in each panel are ordered with the most significant association listed first and in decreasing strength of association with the statistically significant group. Boxplots of each species show the range of relative abundances of the OTU in each grouping. Note that the x-axes scale is different between the panels.

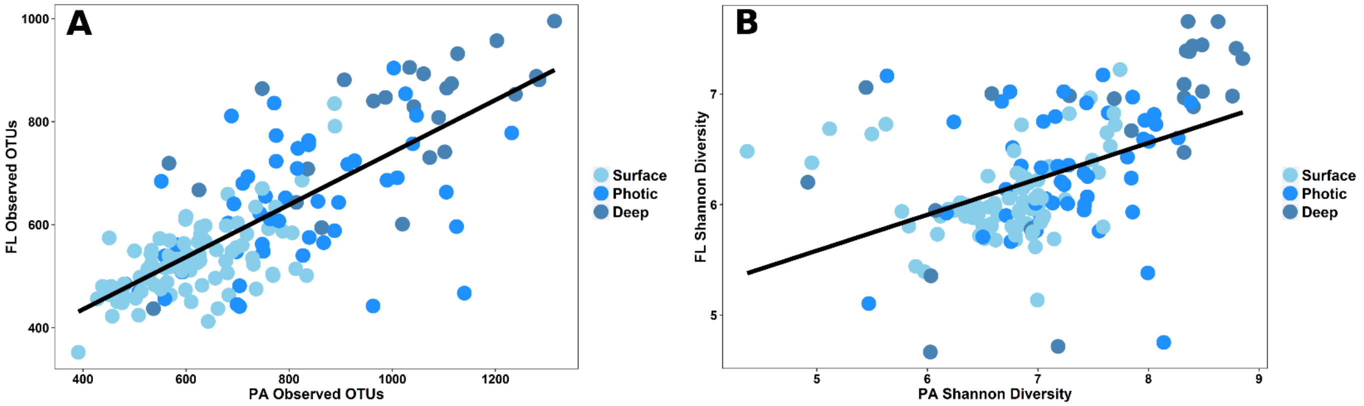

Figure S6. A. Correlation between FL and PA number of observed OTUs. Adjusted $R^2 = 0.5817$, $p < 0.001$ B. Correlation between FL and PA Shannon Diversity. Adjusted $R^2 = 0.2079$, $p < 0.001$. Colours show depth in the water column.

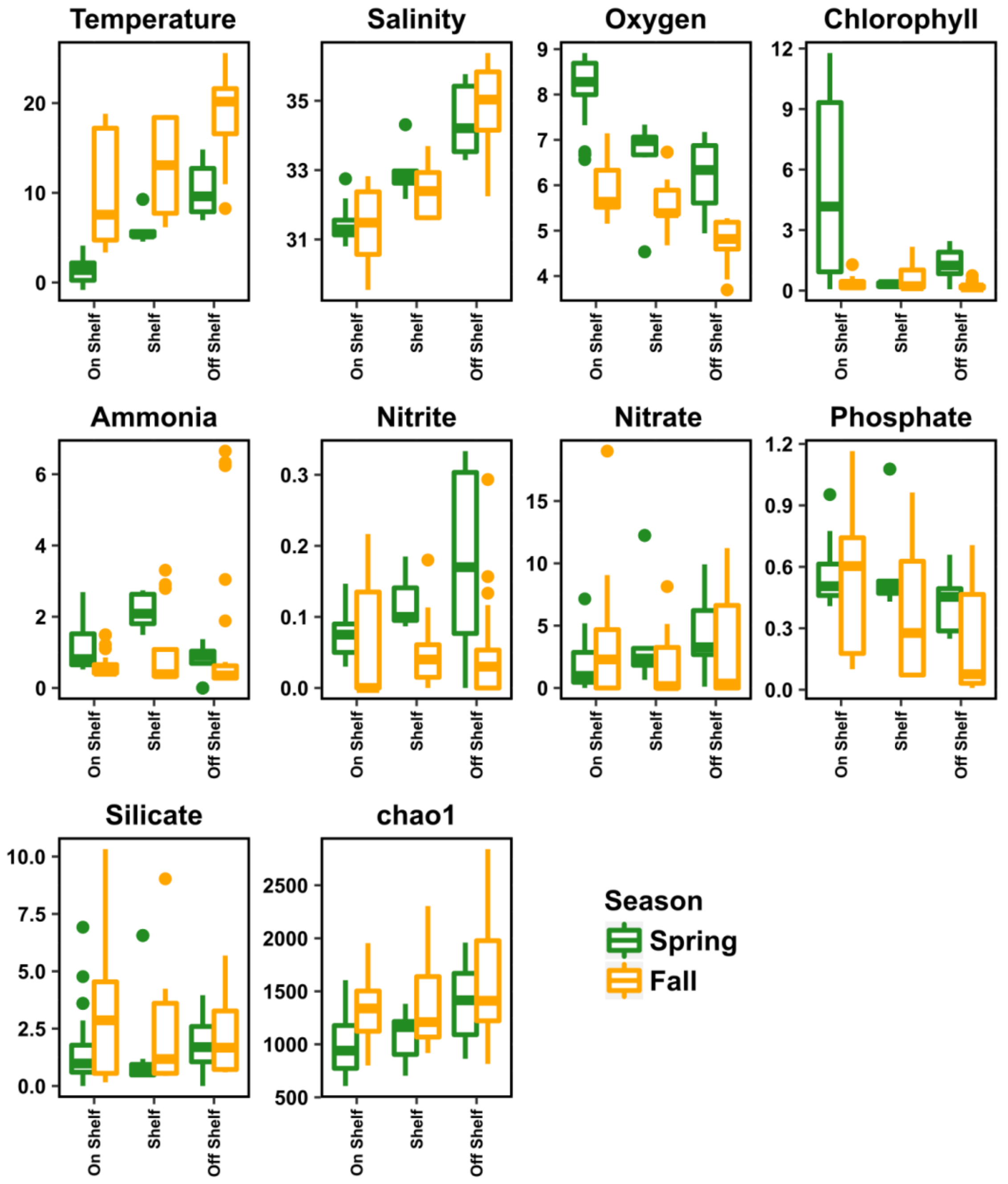

Figure S7. Boxplots showing the ranges of environmental variables in the on shelf, shelf break and off shelf samples (Figure S1) down to 100 m that were used to produce Figure 10. Units are as follows: Salinity (PSU), temperature (°C), oxygen (µmol/kg), chlorophyll *a* (mg/m^3), ammonium (mmol/m^3), nitrite (mmol/m^3), nitrate (mmol/m^3), phosphate (mmol/m^3), silicate (mmol/m^3), chao1 (estimated number of OTUs).

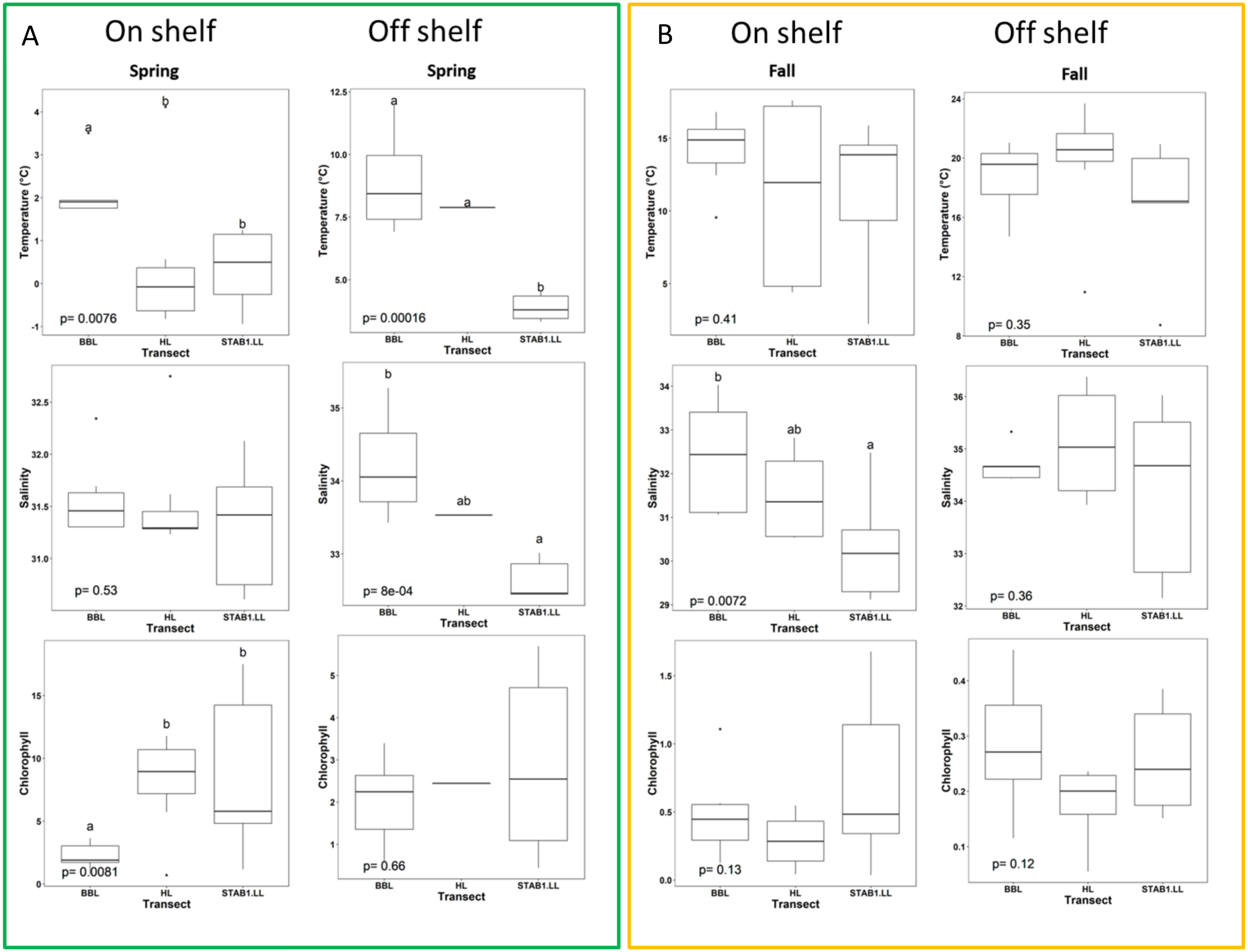
A
On shelf
Off shelf
Spring
Temperature (°C)
p= 0.0076
p= 0.00016
Salinity
p= 0.53
p= 8e-04
Chlorophyll
p= 0.0081
p= 0.66
B
On shelf
Off shelf
Fall
p= 0.41
p= 0.35
p= 0.0072
p= 0.36
p= 0.13
p= 0.12
BBL
HL
STAB1.LL
Transect

Figure S8. Boxplots of the Temperature (°C), Salinity (PSU) and Chlorophyll a concentrations (mg/m^3), in on shelf and off shelf sections of the BBL, HL and STAB transects in A) spring and B) fall cruises.

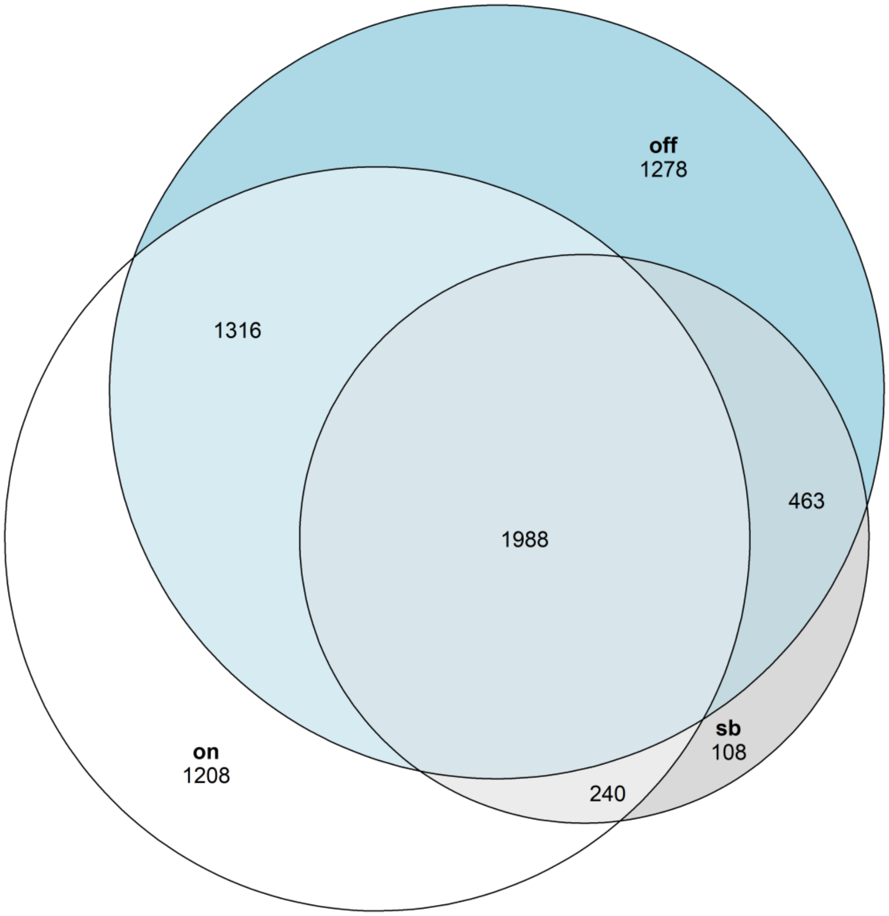

% shared OTUs	Shelf Break (sb)	on-shelf (on)	off-shelf (off)
All	71.0	41.8	39.4
on-shelf/shelf break	79.6	46.9	N/A
off-shelf/shelf break	87.6	N/A	48.6
off-shelf/on-shelf	N/A	69.5	65.5
Unique	3.9	25.4	25.3

Figure S9. Venn diagram showing the number of shared OTUs between the on-shelf, off-shelf and shelf break regions. The table below shows the percentage of shared OTUs out of the total number of OTUs for that region.

Supplementary Tables

Table S1. List of stations and depth sampled along the Halifax Line (HL) during 2014 and 2016 Spring and Fall missions. Y indicates that the station (and depth) was sampled and N indicates that it was not sampled.

Station	Latitude	Latitude2	Depths	2014 Spring	2014 Fall	2016 Spring	2016 Fall
HL1	44.4005	-63.4499	1	Y	Y	Y	Y
			20	Y	Y	Y	Y
			40	Y	Y	Y	Y
			60	Y	Y	Y	Y
HL2	44.2664	-63.3169	1	Y	Y	Y	Y
			20	Y	Y	Y	Y
			40	Y	Y	Y	Y
			80	Y	Y	Y	Y
HL4	43.4813	-62.4541	1	Y	Y	Y	Y
			20	Y	Y	Y	Y
			40	Y	Y	Y	Y
			60	Y	Y	Y	Y
HL5.5	42.9396	-61.8342	1	Y	N	Y	Y
			20	Y	N	Y	Y
			80	Y	N	Y	Y
			250	Y	N	Y	Y
HL6	42.8321	-61.7324	1	N	Y	Y	Y
			20	N	Y	Y	Y
			50	N	N	Y	Y
			80	N	Y	Y	Y
			250	N	Y	N	N
HL7	42.4763	-61.4359	1	N	N	Y	Y
			20	N	N	Y	Y
			50	N	N	Y	Y
			80	N	N	Y	Y
HL8	42.3623	-61.3384	1	Y	Y	N	Y
			20	Y	Y	N	N
			60	N	N	N	Y
			100	Y	Y	N	Y
			250	Y	Y	N	Y
HL11	41.7778	-60.9077	1	N	Y	N	Y
			20	N	Y	N	N
			45	N	N	N	Y
			80	N	Y	N	N
			100	N	N	N	Y
			250	N	Y	N	Y
HL2Ret	44.2664	-63.3169	1	N	N	Y	Y
			20	N	N	Y	Y
			40	N	N	Y	Y
			80	N	N	Y	Y

Table S2. List of stations describing sampling depth, geographical coordinates and physiochemical characteristics of the water samples from 2014. (Excel file)

Table S3. List of dual-indexing Illumina fusion primers used for Multiplexing samples for Illumina Sequencing (Excel file)

Table S4. List of dominant OTUs overall and in spring and fall

Taxon (OTU#)	fraction	Overall (%)	Spring (%)	Fall (%)
Pseudoalteromonas sp. OTU#827726),	FL			
Pelagibacteraceae (OTU#637092)	FL	13	16	
Pelagibacteraceae (OTU#307744)	FL	6		
Rhodobacteraceae (OTU#569286)	FL	4		
Pseudoalteromonas sp. OTU#827726,	FL		4	
Rhodobacteraceae sp. OTU#804483	FL		3	
Pelagibacteraceae OTU#307744	FL			10
Pelagibacteraceae OTU#637092	FL			10
Rhodobacteraceae sp. OTU#569286	FL			7
Alteromonas sp. OTU#565946	PA	4		
Ulvibacter sp. OTU#774258	PA	3		
OM60 OTU#630330	PA	3	4	
Ulvibacter sp. OTU#774258	PA		6	
Flavobacteriaceae sp. OTU#659486	PA		4	
Alteromonas sp. OTU#565946	PA			8
Rhodobacteraceae (OTU#569286	PA			4
Synechococcus (OTU#831789	PA			4
Erythrobacteraceae (OTU#582344)	PA			4

Table S5. Detailed list of indicator species for various sample classifying categories including size, season, depths, and location on the shelf. Each test is a separate tab. (Excel file)

Table S6. Summary of indicator species for size fraction, season, and depth

Taxon (OTU)	fraction	season	depth
Fluviicola (Cryomorphaceae) (OTU#228555)	PA		
OM60 (OTU#630330)	PA		
Pelagibacteraceae (OTU#637092)	FL		
Rhodobacteraceae (OTU#534690)		Fall	
Rhodobacteraceae (OTU#574650)		Fall	
Synechococcus (OTU#831789)		Fall	
Synechococcus (OTU#404788)		Fall	
Flavobacterium sp. (OTU#689465)		spring	
Ulvibacter sp. (OTU#774258)		spring	
Cryomorphaceae sp. (OTU#666579)		Spring	
Rhodobacteraceae (OTU#272142)		Spring	
Rhodobacteraceae (OTU#645011)		Spring	
Thiohalorhabdales (OTU#833015)			deep
Flavobacteriaceae sp. (OTU#636896)			Surface
Alphaproteobacteria sp. (OTU 335943)			Surface
Rhodobacteraceae sp. (OTU#804483)			Surface
Synechococcus sp. (OTU#404788)			Surface

Table S7. Welch's T-tests comparing differences between environmental parameters of on shelf and off shelf samples. Tests only include stations above 100 m depth. Variables that are significantly different between on and off shelf samples are marked in bold.

Variable	On shelf mean ± SD	Off shelf mean ± SD	*p value*
**Temperature**	**6.04 ± 6.65**	**15.63 ± 5.67**	**1 e^{-12}**
**Salinity**	**31.42 ± 0.77**	**34.73 ± 1.08**	**2 e^{-16}**
**Oxygen**	**6.97 ± 1.28**	**5.34 ± 0.91**	**2 e^{-11}**
**Chlorophyll**	**2.71 ± 3.84**	**0.65 ± 0.75**	**0.0003**
Ammonia	0.84 ± 0.51	0.98 ± 1.45	0.5
Nitrite	0.067 ± 0.06	0.098 ± 0.11	0.07
Nitrate	2.65 ± 3.56	3.46 ± 3.63	0.2
**Phosphate**	**0.53 ± 0.27**	**0.31 ± 0.24**	**3 e^{-5}**
Silicate	2.54 ± 2.87	2.01 ± 1.42	0.2

Table S8. List and presence (+) and absence (blank) of the dominant taxa and their associated size fraction (FL for free living and PA particle associated) in the Spring and Fall, on-shelf and off-shelf regions

	Fraction	Spring		Fall	
**Taxa**		**on**	**off**	**on**	**off**
Pelagibacter **(OTU637092)**	FL	+	+	+	+
Alteromonas **(OTU565946)**	PA	+	+	+	+
**Rhodobacteriaceae (OTU569286)**	FL	+	+	+	+
Polaribacter **(OTU28929)**		+			
**Alteromonadales (OTU535135)**		+			
Octadecabacter **(OTU272142)**		+			
**Rhodobacteriaceae (OTU645011)**		+			
**Colwelliaceae (OTU583907)**		+			
Ulvibacter **(OTU774258)**	PA	+	+		
**Rhodobacteriaceae (OTU785501)**		+	+		
Octadecabacter **(OTU804483)**		+	+	+	
Octadecabacter **(OTU804449)**		+	+	+	
**Oceanospirillales (OTU1712364)**		+	+	+	
**Rhodobacteriaceae (OTU714708)**		+	+	+	
Pseudoalteromonas **(OTU827726)**	FL	+	+		+
Alteromonas **(OTU580254)**			+	+	+
**Pelagibacteriaceae (OTU307744)**	FL		+	+	+
**Pelagibacteriaceae (OTUC)**	FL		+	+	
**SAR86 (OTU564740)**			+		
**Pelagibacteriaceae (OTU320187)**	FL		+		+
**Pelagibacteriaceae (OTU556042)**	FL			+	+
**Pelagibacteriaceae (OTU352844)**	FL			+	
Synechococcus **(OTU404788)**				+	
Synechococcus **(OTU831789)**				+	
**Rhodobacteraceae (OTU534690)***				+	
**Erythrobacteraceae (OTU582344)**					+
Prochlorococcus **(OTU227663)**	FL				+
Pelagibacter **(OTU343006)**	FL				+
Methylobacterium **(OTU542475)**					+

Table S9. ANOSIM test results (9999 permutations) determining significance of seasonality, size fraction, and depth on community structure (Bray-Curtis similarity) for all samples from 2014.

**Grouping**	**ANOSIM statistic R**	***p***
**Season**	0.4655	0.0001
**Size**	0.3732	0.0001
**Depth**	0.2142	0.0001
